# Novel function of PiT1/SLC20A1 in LPS-related inflammation and wound healing

**DOI:** 10.1038/s41598-018-37551-1

**Published:** 2019-02-12

**Authors:** Eugénie Koumakis, Joëlle Millet-Botti, Jamel El Benna, Christine Leroy, Valérie Boitez, Patrice Codogno, Gérard Friedlander, Anne Forand

**Affiliations:** 1 INSERM UMR_S1151 CNRS UMR8253 Institut Necker-Enfants Malades (INEM) Université Paris Descartes, Paris, France; 2 Rheumatology Department, Cochin Hospital, APHP, Paris, France; 3 Centre de Référence des Maladies Rares du Métabolisme du Calcium et du Phosphate, site constitutif, Cochin Hospital, Paris, France; 4 Université Paris Diderot-Sorbonne Paris Cité, F-75993 Paris, France; 5 INSERM U1149, CNRS-ERL8252, Centre de Recherche sur l’Inflammation, Université Paris Diderot, Sorbonne Paris Cité, Laboratoire d’Excellence Inflamex, Faculté de Médecine, Site Xavier Bichat, 75018 Paris, France; 6 Inovarion, Paris, France

## Abstract

PiT1/SLC20A1 is an inorganic phosphate transporter with additional functions including the regulation of TNFα-induced apoptosis, erythropoiesis, cell proliferation and insulin signaling. Recent data suggest a relationship between PiT1 and NF-κB-dependent inflammation: (i) *Pit1* mRNA is up-regulated in the context of NF-κB pathway activation; (ii) NF-κB target gene transcription is decreased in PiT1-deficient conditions. This led us to investigate the role of PiT1 in lipopolysaccharide (LPS)-induced inflammation. MCP-1 and IL-6 concentrations were impaired in PiT1-deficient bone marrow derived macrophages (BMDMs) upon LPS stimulation. Lower MCP-1 and IL-6 serum levels were observed in *Mx1-Cre*; *Pit1*^*lox*/*lox*^ mice dosed intraperitoneally with LPS. Lower PiT1 expression correlated with decreased *in vitro* wound healing and lower reactive oxygen species levels. Reduced IκB degradation and lower p65 nuclear translocation were observed in PiT1-deficient cells stimulated with LPS. Conversely, PiT1 expression was induced *in vitro* upon LPS stimulation. Addition of an NF-κB inhibitor abolished LPS-induced PiT1 expression. Furthermore, we showed that p65 expression activated *Pit1* promoter activity. Finally, ChIP assays demonstrated that p65 directly binds to the *mPit1* promoter in response to LPS. These data demonstrate a completely novel function of PiT1 in the response to LPS and provide mechanistic insights into the regulation of PiT1 expression by NF-κB.

## Introduction

PiT1 (also known as SLC20A1) and PiT2 (also known as SLC20A2) were originally identified as mammalian retrovirus receptors, but it was soon discovered that they function as sodium-dependent importers of inorganic phosphate (Pi)^1–3^. *Pit1* and *Pit2* mRNAs are expressed in most tissues and organs, and so these transporters were assumed to have a housekeeping, possibly redundant, function in Pi homeostasis^1,2^. The absence of redundancy in the functions of PiT1 and PiT2 proteins was demonstrated *in vivo* with the deletion of the *Pit1* gene in mice^4,5^. The complete knock out (KO) of *Pit1* results in an embryonic lethal phenotype, despite an increase in the *Pit2* mRNA levels^4^. PiT1 also has specific functions in some tissues and cell types; for example, it is involved in pathological vascular calcifications^6^ and in the proliferation and differentiation of osteoblasts and chondrocytes^7,8^. Additionally, novel functions of PiT1 have recently been identified. PiT1 is involved in the regulation of cell proliferation, density, and adhesion^9–11^, liver development^4^, TNFα-induced apoptosis^12^, and erythroid and B cell differentiation^13,14^. Our group has recently discovered that PiT1 also plays a role in regulating metabolism^15^. Specific *Pit1* KO in hepatocytes significantly improves glucose tolerance and insulin sensitivity, enhances insulin signaling, and decreases hepatic lipogenesis^15^. We also showed that PiT1-deficient mice are protected against high fat diet-induced obesity and diabetes.

Importantly, several observations from our group and others point toward a link between PiT1 and the transcription factor NF-κB. Firstly, the *Pit1*-KO phenotype is strongly reminiscent of the one displayed by embryos in which NF-κB signaling pathway components have been knocked out^16–19^. Secondly, *Pit1* transcription is strongly upregulated early following partial hepatectomy^4,20^, during the so-called “priming phase” of liver regeneration, which is dependent on the rapid activation of the NF-κB pathway and the subsequent transcription of NF-κB target pro-inflammatory genes such as *Tnfα* and *Il-6*^16,20,21^, and other studies indicate that *PiT1* expression is regulated by induced or basal activity of NF-κB^22–24^. Moreover, *Pit1* mRNA levels are increased in the livers of mice when the NF-κB pathway is upregulated due to the deletion of one of its regulators, the Von Hippel-Lindau protein (pVHL)^24^. Thirdly, our group has recently investigated the role of PiT1 in liver regeneration *in vivo* using the model of liver regeneration following 2/3^rd^ hepatectomy (PH). During the first hours following PH, mice heterozygous for a deletion in *Pit1* (*Pit1*^+/∆5^) had lower hepatic *Il-6* mRNA levels and lower serum IL-6 compared to control mice. *IL-6* is a known NF-κB target gene. Mice with liver-specific *Pit1* deletion (the *Alb-Cre*; *Pit1*^*lox*/*lox*^ mice) had normal cytokine production during this phase (unpublished data). This led us to hypothesize that the impairment in cytokine production in *Pit1*^+/∆5^ mice may be due to lack of PiT1 in macrophages rather than in hepatocytes.

NF-κB is an inducible transcription factor^25^. Since its discovery in 1986^26^, the NF-κB pathway has been shown to be involved in multiple biological functions including cell adhesion, differentiation, proliferation^27^, autophagy, senescence^28,29^, and protection against apoptosis^30^. The most important and evolutionarily conserved role of NF-κB is as a mediator of the immune and inflammatory response^25^. Considering these data, and to elucidate novel aspects of PiT1 function, we sought to investigate whether PiT1 plays a role in the NF-κB-mediated inflammatory response in macrophages and to examine PiT1 regulation by NF-κB.

## Results

### PiT1 depletion is associated with lower *Mcp-1* mRNA and MCP-1 protein levels *in vitro*

**Figure 1:**
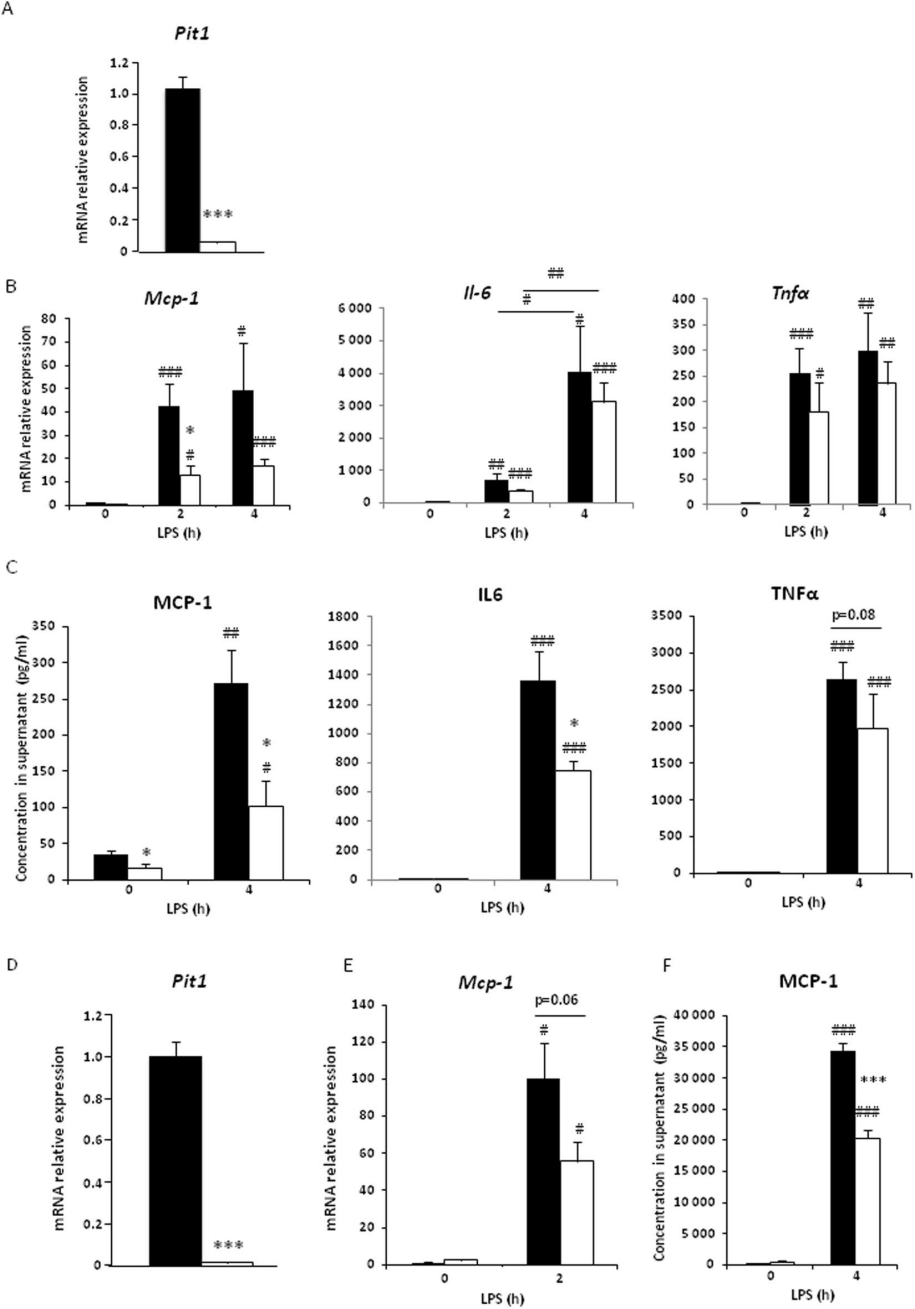
PiT1 depletion is associated with lower *Mcp-1* mRNA and MCP-1 protein levels *in vitro*. (**A**) RT-qPCR analysis of *Pit1* mRNA expression in BMDMs from *Mx1-Cre*; *Pit1*^*lox*/*lox*^ mice (white bars) and control mice (black bars). Data were normalized to data from control cells. Data are means ± S.E.M. of at least three independent experiments. (**B**) RT-qPCR analysis of *Mcp-1*, *Il-6*, and *Tnfα* expression in BMDMs from *Mx1-Cre*; *Pit1*^*lox*/*lox*^ mice (white bars) and controls (black bars) stimulated *in vitro* with 10 ng/ml LPS for the indicated times. Data were normalized to data from non-stimulated control cells. Data are means ± S.E.M. of at least three independent experiments. (**C**) Quantification by ELISA of the indicated cytokines and chemokines in BMDM supernatants from *Mx1-Cre*; *Pit1*^*lox*/*lox*^ (white bars) and control mice (black bars) stimulated *in vitro* with 10 ng/ml LPS for the indicated times. Data are means ± S.E.M. of at least three independent experiments. (**D**) RT-qPCR analysis of *Pit1* mRNA expression in WT MEFs (black bars) and *Pit1-*KO MEFs (white bars). Data were normalized to data from WT cells. Data are means ± S.E.M. of at least three independent experiments. (**E**) RT-qPCR analysis of *Mcp-1* mRNA expression and (**F**) ELISA quantification of MCP-1 in the supernatant of WT MEFs (black bars) and *Pit1*-KO MEFs (white bars) stimulated with 100 ng/ml LPS for the indicated times. Data are means ± S.E.M. of at least three independent experiments. Student’s unpaired t-test or an Unpaired t-test with Welch correction for groups with unequal variance was performed; ^#^indicates comparison with the untreated condition; *indicates comparison between control and PiT1-deficient cells; *p < 0.05; **p < 0.01; ***p < 0.001; ^#^p < 0.05; ^##^p < 0.01; ^###^p < 0.001.

Murine bone marrow-derived macrophages (BMDMs) were obtained from *Mx1-Cre*; *Pit1*^*lox*/*lox*^ and control mice. Mean *Pit1* mRNA levels in macrophages, as assessed by RT-qPCR, were reduced by 94.3% ± 0.7 (80 to 98%) in the *Mx1-Cre*; *Pit1*^*lox*/*lox*^ mice compared to the controls (Fig. 1A). The mRNA expression and supernatant concentrations of cytokines and chemokines known to be induced by LPS were studied before and after LPS stimulation of the BMDMs for the indicated times. PiT1-deficient macrophages had lower levels of *Mcp-1* mRNA (Fig. 1B), and the MCP-1 protein concentration in the supernatant of PiT1-deficient macrophages was lower than in the supernatant of control macrophages following stimulation with 10 ng/ml LPS (Figs 1C and S1D). IL-6 protein levels were also significantly lower in supernatants of PiT1-deficient BMDMs after LPS stimulation than in controls (Figs 1C and S1E). Although not significant, similar decreases after LPS treatment were observed for *Tnfα* and *Il-6* mRNA levels between PiT1-deficient and control BMDMs (Figs 1B and S1B,C). In order to exclude the possibility that our results were caused by a differential expression of LPS receptor TLR4 between PiT1-deficient and control cells, *Tlr4* mRNA expression was evaluated and no difference was observed (Fig. S2).

We also studied the LPS-induced cytokine and chemokine response in mouse embryonic fibroblasts (MEFs) isolated from wild-type (WT) and *Pit1*-KO embryos. MEFs were chosen because these cells do not express endogenous *Pit1* due to the gene deletion (Fig. 1D) and because they express toll-like receptors (TLR) and thus are able to transduce LPS-TLR signaling^31^. As observed in BMDMs, a lower MCP-1 protein concentration was observed in the supernatant of *Pit1*-KO MEFs following LPS stimulation than in WT cells (Figs 1F and S1H). Although *Tnfα* and *Il-6* mRNA levels appeared to be lower after stimulation in *Pit1*-KO cells, these results were not significant (Fig. S3A,B) and no differences in TNFα or IL-6 concentrations were observed (Fig. S3C,D). Taken together, these results suggest that PiT1 depletion decreases LPS-induced MCP-1 concentration *in vitro*.

Vigorous production of pro-inflammatory cytokines such as IL-6 and TNFα is a hallmark of classically activated macrophages, also known as effector or M1 macrophages^32^. We next investigated whether PiT1 depletion could also be responsible for a modification in M2 phenotype. M2 gene *Il-10* was expressed at a significantly lower level upon 2 h LPS stimulation in PiT1-deficient BMDMs than in control BMDMs, suggesting an effect of PiT1 depletion on M2 gene expression (Fig. S4).

### Loss of PiT1 modulates LPS response *in vivo*

**Figure 2:**
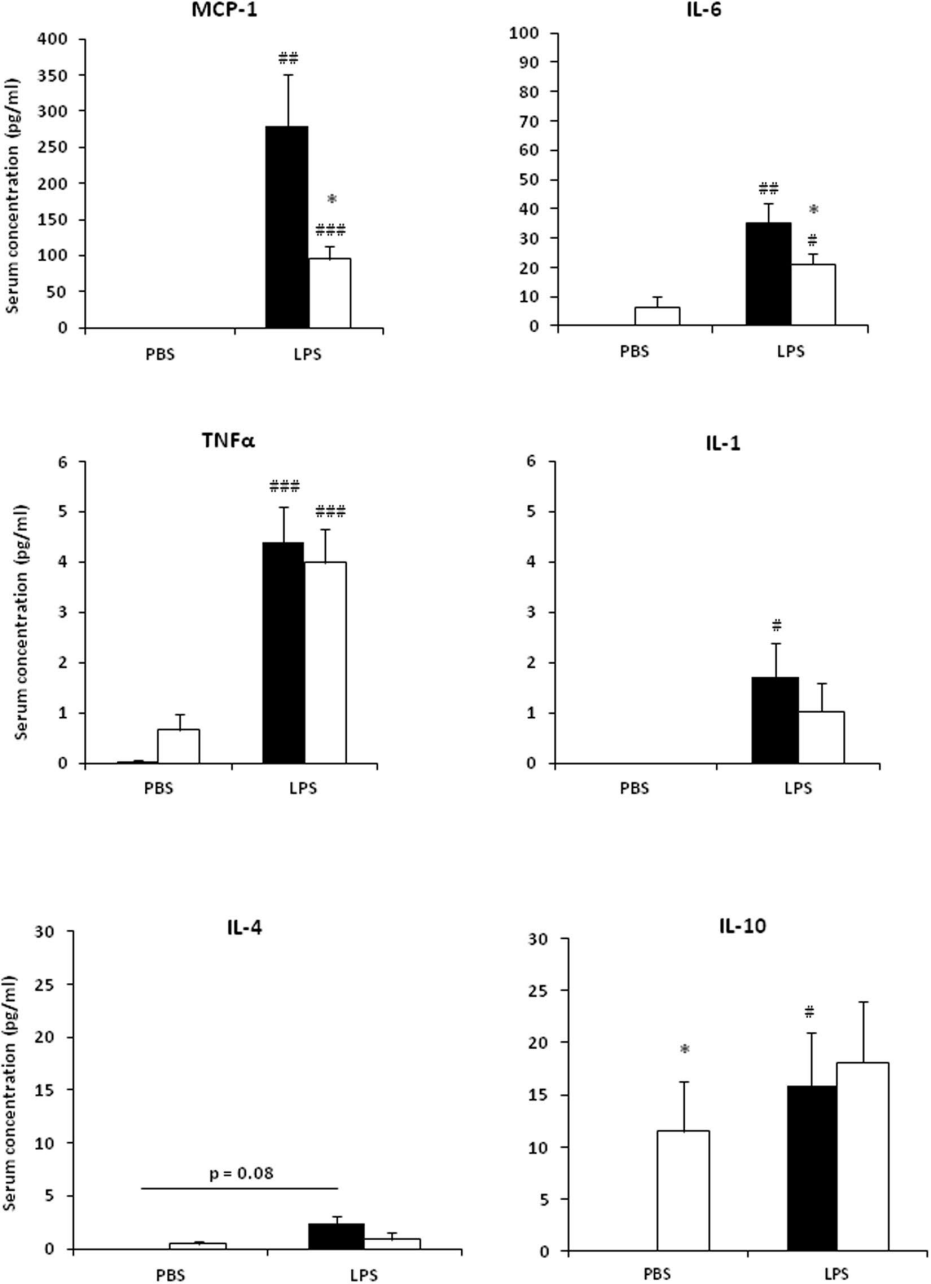
Loss of PiT1 modulates LPS response *in vivo*. Multiplex ELISA of serum cytokines 24 h after intraperitoneal injection of LPS (500 ng/g body weight) into 16 week-old male *Mx1-Cre*; *Pit1*^*lox*/*lox*^ (white bars) and control mice (black bars). Data are the means ± S.E.M. of at least 10 mice per group. Student’s unpaired t-test or an Unpaired t-test with Welch correction for groups with unequal variance was performed; ^#^indicates comparison with the untreated condition; *indicates comparison between control and *Mx1-Cre*; *Pit1*^*lox*/*lox*^ mice; *p < 0.05; ^#^p < 0.05; ^##^p < 0.01; ^###^p < 0.001.

Considering the lower overall LPS-induced inflammatory response observed in PiT1-deficient BMDMs and *Pit1*-KO MEFs, we sought to investigate the effects of PiT1 deficiency *in vivo*. For this purpose, we performed intraperitoneal injections of LPS (0.5 µg/g) or, as a control, PBS at 24 h before sacrifice of 16 week-old *Mx1-Cre*; *Pit1*^*lox*/*lox*^ and control mice. As expected, LPS injection caused an increase in MCP-1, IL-6, and TNFα serum concentrations. As observed *in vitro* in BMDMs, serum levels of MCP-1 and IL-6 were significantly lower in *Mx1-Cre*; *Pit1*^*lox*/*lox*^ than in control mice after LPS injection (Fig. 2). No significant differences were observed for TNFα and IL-1 concentrations between *Mx1-Cre*; *Pit1*^*lox*/*lox*^ and control mice. Serum concentrations of IL-4, classically described as a M2-activating cytokine, were not significantly different between *Mx1-Cre*; *Pit1*^*lox*/*lox*^ and control mice. The baseline serum IL-10 level was significantly higher in *Mx1-Cre*; *Pit1*^*lox*/*lox*^ mice than in control mice; however, no difference was observed between *Mx1-Cre*; *Pit1*^*lox*/*lox*^ and control mice in the LPS-stimulated condition. These results show impaired concentrations of MCP-1 and IL-6 following LPS stimulation in the absence of PiT1.

### PiT1 influences macrophage function

#### Wound healing *in vitro*

**Figure 3:**
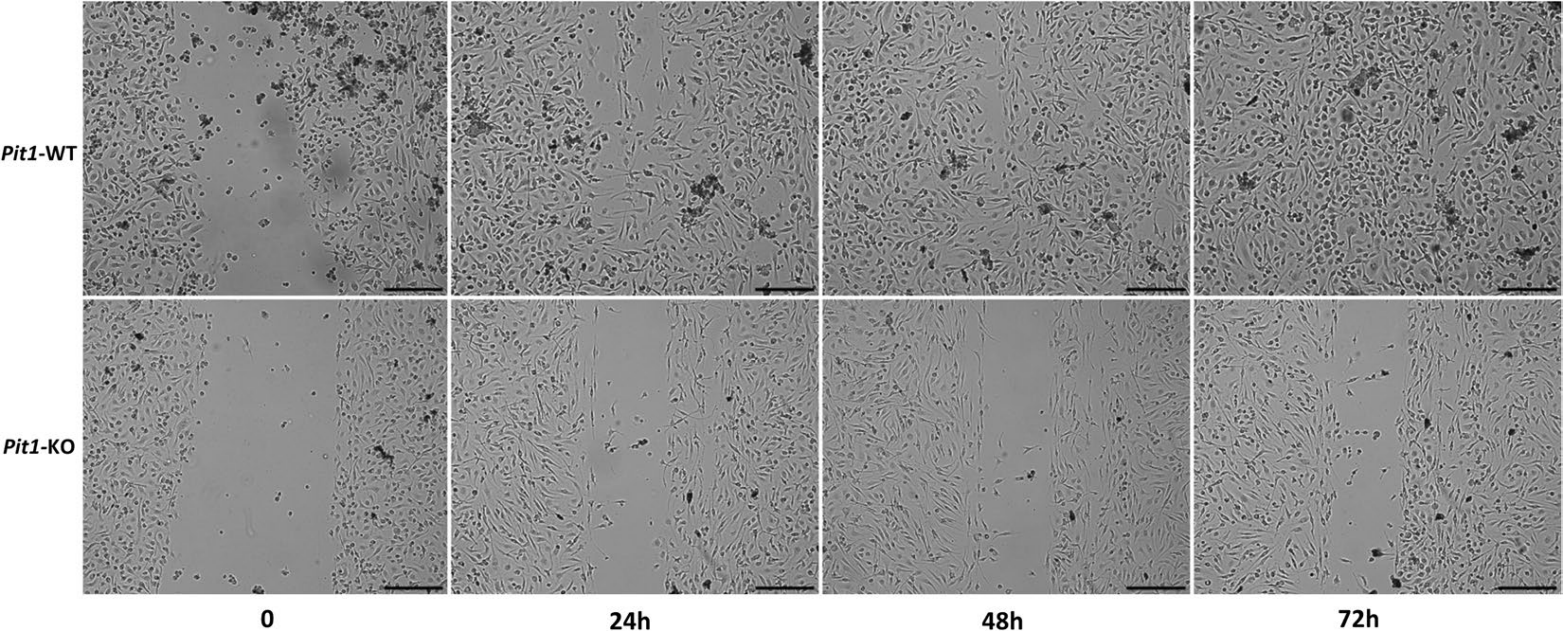
PiT1-deficient cells show impaired wound healing. Representative pictures of wound healing assay. BMDMs from *Mx1-Cre*; *Pit1*^*lox*/*lox*^ (lower panel) and control mice (upper panel) were seeded on an IBIDI µ-slide 8-well, glass-bottom plates with culture inserts. After 72 h, IBIDI inserts were removed. Closure of the resulting wound was photographed every 24 h over the next 72 h with a 10x objective (scale bar = 300 µm).

MCP-1 plays a major role in the chemo-attraction of monocytes and macrophages during inflammatory conditions^33^ and is known to contribute to wound healing^34^. We therefore assayed the effects of PiT1 depletion on the migratory and chemoattractant abilities of macrophages *in vitro*. Wounds generated in PiT1-deficient BMDMs healed more slowly and incompletely than wounds across control BMDM cultures (Fig. 3). This result suggests that PiT1 deficiency has functional consequences on macrophage migration and wound repair abilities perhaps by modulating MCP-1 concentration.

#### Thioglycollate-induced peritonitis

MCP-1 is necessary for the recruitment of monocytes in several models of experimental peritonitis^35^. Thioglycollate-induced peritonitis is characterized by substantial inflammation and accumulation of inflammatory macrophages^36^. MCP-1 is the primary chemokine required for monocyte recruitment in mouse peritonitis induced with thioglycollate, and the induction of endogenous MCP-1 in this system is highly macrophage-dependent^35^. Peritonitis was induced by injection of 1 ml of 4% thioglycollate brewer medium into the peritoneal cavities of 12–16 week-old mice; controls were dosed with PBS. After 72 h, monocyte peritoneal infiltration was analyzed. The number of cells recruited after thioglycollate injection was significantly higher than after PBS injection (data not shown). We then quantified and sorted peritoneal macrophages by flow cytometry and identified resident and recruited macrophages, which differ by the intensity of CD11b fluorescence. No difference was observed in the number of recruited macrophages between *Mx1-Cre*; *Pit1*^*lox*/*lox*^ and control mice after thioglycollate injection (Fig. S5).

### PiT1 depletion decreases reactive oxygen species (ROS) production

**Figure 4:**
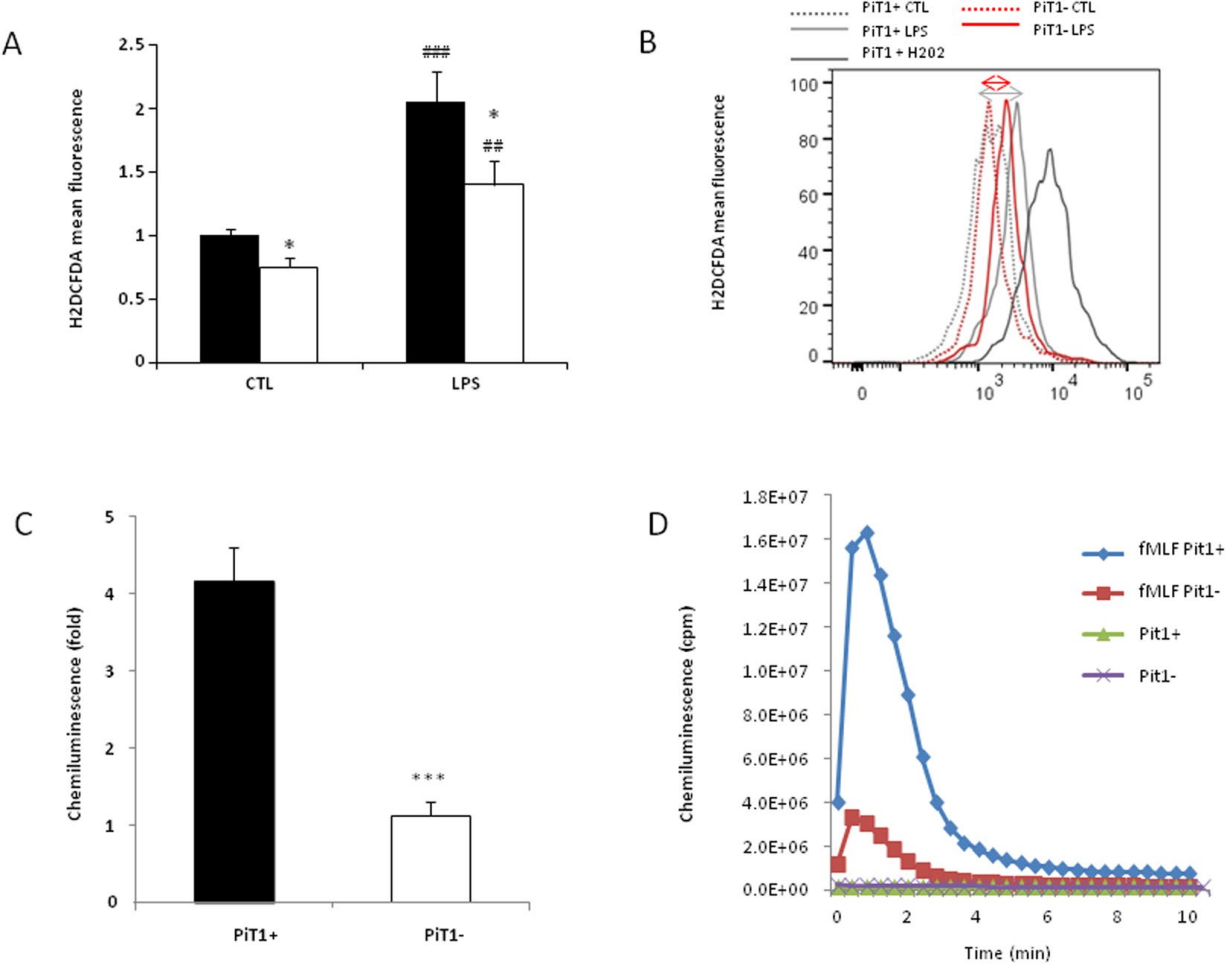
PiT1-deficient cells show impaired ROS production. (**A**,**B**) Measurement of intracellular ROS production upon LPS treatment using CM-H2DCFDA probe. (**A**) Flow cytometry analysis of CM-H2DCFDA fluorescence in BMDMs from *Mx1-Cre*; *Pit1*^*lox*/*lox*^, i.e., PiT1-deficient cells (white bars) and controls (black bars) following 7-day differentiation. Macrophages were treated with complete medium with or without 1000 ng/ml LPS for 20 h. Each bar represents means ± S.E.M. of three independent experiments including BMDMs from at least three *Mx1-Cre*; *Pit1*^*lox*/*lox*^ and three control mice. ^#^Indicates comparison to untreated cells; ^##^p < 0.01, ^###^p < 0.001; *indicates p < 0.05 as compared to control cells. Student’s unpaired t-test or an Unpaired t-test with Welch correction for groups with unequal variance was performed (**B**) Data from a representative CM-H2DCFDA fluorescence analysis. (**C**,**D**) Measurement of ROS production by luminol-amplified chemiluminescence. (**C**) BMDMs from *Mx1-Cre*; *Pit1*^*lox*/*lox*^, i.e., PiT1-deficient cells (white bar) and control mice (black bars) following 7-day differentiation were harvested in HBSS and stimulated with fMLF at a final concentration of 10^−5^ M in presence of luminol and peroxidase. Chemiluminescence was directly quantified and given as the ratio between fMLF-treated and basal conditions. Data are means ± S.E.M of at least three independent experiments. Student’s unpaired t-test or an Unpaired t-test with Welch correction for groups with unequal variance was performed *indicates comparison with control cells; ***p < 0.001. (**D**) Representative chemiluminescence experiment.

Inflammatory stimulants such as LPS induce the generation of ROS in macrophages^37–39^. ROS are involved in bacterial killing and cytokine production. When we stimulated BMDMs with 1000 ng/ml LPS hydrogen peroxide (H_2_O_2_) production was higher than in unstimulated cells after 20 h as assessed using the general oxidative stress indicator CM-H2DCFDA. In comparison with control BMDMs, the production of H_2_O_2_ in PiT1-deficient BMDMs was significantly lower (Fig. 4A,B), suggesting that PiT1 contributes to ROS production.

NOX2 is the NADPH oxidase primarily expressed in macrophages; therefore, we next examined whether PiT1 deficiency correlates with differences in NOX2 activity. NOX2 activation in macrophages was assessed using the bacterial peptide formyl-methionyl-leucyl-phenylalanine (fMLF) to stimulate the cells and a luminol plus HRP-amplified chemiluminescence assay to monitor NOX2 activation. We found that ROS production by PiT1-deficient BMDMs was dramatically decreased compared to BMDMs from control mice (Fig. 4C,D). As ROS are known to participate in killing of pathogens after phagocytosis^40^, we also investigated whether phagocytosis was altered in PiT1-deficient BMDMs. No difference was observed in the phagocytic abilities of PiT1-deficient and control BMDMs (Fig. S6), suggesting that although ROS production is lower in the absence of PiT1, this does not necessarily impair the phagocytosis process *in vitro*.

### LPS-induced PiT1 expression correlates with NF-κB target gene induction

**Figure 5:**
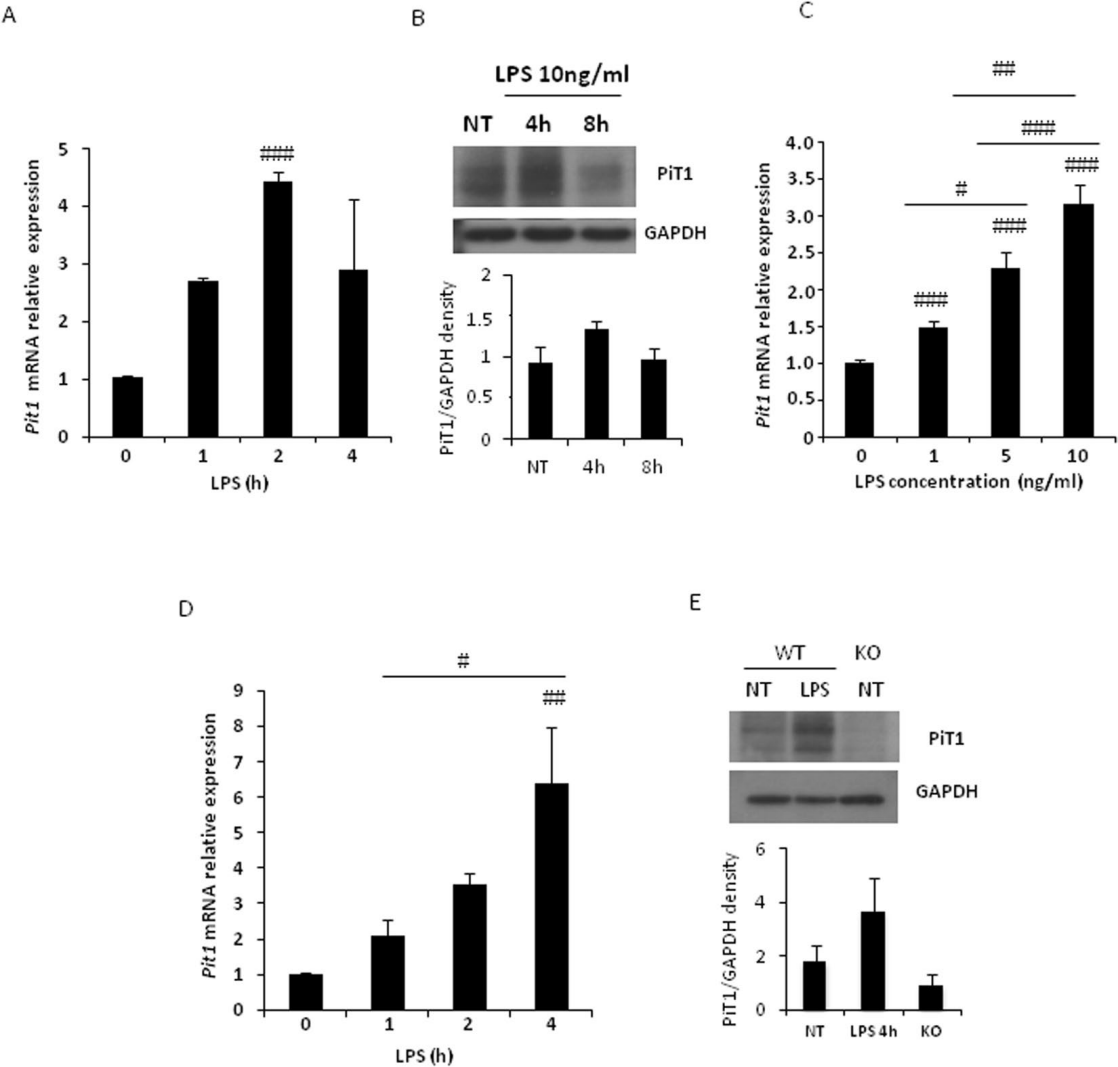
PiT1 expression is induced by LPS in a dose-dependent manner. (**A**) RT-qPCR analysis of *Pit1* mRNA expression in control BMDMs stimulated *in vitro* with 10 ng/ml LPS for the indicated times. Results were normalized to data from non-stimulated control cells. Data are means ± S.E.M. of three independent experiments. ANOVA test and Tukey’s multiple comparison test were performed. ^#^Indicated comparison with untreated condition, ^###^p < 0.001. (**B**) Representative western blot of PiT1 in BMDMs following treatment with 10 ng/ml LPS for the indicated times, with the corresponding quantification. (**C**) RT-qPCR analysis of *Pit1* mRNA expression in control BMDMs stimulated *in vitro* with the indicated concentration of LPS for 16 h. Results were normalized to data from non-stimulated cells. Data are means ± S.E.M. of three independent experiments. ANOVA test and Tukey’s multiple comparison test were performed. ^#^p < 0.05; ^##^p < 0.01; ^###^p < 0.001. (**D**) RT-qPCR analysis of *Pit1* mRNA expression in WT MEFs following treatment with 100 ng/ml LPS treatment. Results were normalized to data from non-stimulated cells. Data are means ± S.E.M. of three independent experiments. ANOVA test and Tukey’s multiple comparison test were performed. ^#^p < 0.05; ^##^p < 0.01. (**E**) Representative western blot of PiT1 in MEFs following treatment with 100 ng/ml LPS for 4 h. Full length blots are presented in Supplementary Fig. 9.

We next sought to investigate the consequence of LPS stimulation on PiT1 expression. Similar to the increase in *Mcp-1*, *Il-6*, and *Tnfα*, *Pit1* mRNA and PiT1 protein expression increased in BMDMs upon LPS stimulation (Fig. 5A,B). The LPS-induced *Pit1* expression occurred rapidly (Fig. 5A) in the same time frame as increases in *Mcp-1*, *Il-6*, and *Tnfα* mRNAs and was LPS dose-dependent (Fig. 5C). Similar results were found in MEFs (Fig. 5D,E). These findings show that PiT1 expression is induced by LPS treatment and suggest that *Pit1* gene expression might be regulated by transcription factors involved in the LPS-TLR pathway.

In contrast to the effect of LPS on *Pit1* mRNA expression, we did not observe any LPS effect on *Pit2* mRNA expression in control BMDMs (Fig. S7). Interestingly, *Pit2* mRNA expression tended to increase in PiT1-deficient BMDMs following LPS stimulation, and significantly higher *Pit2* expression was observed in PiT1-deficient cells compared to control BMDMs following LPS 4 h stimulation, suggesting a possible compensatory effect for the absence of PiT1. Nevertheless, this possible compensation did not prevent the effects of PiT1 depletion on MCP-1 and IL-6 levels indicative of a PiT1-specific response to LPS.

### LPS-induced *Pit1* expression in macrophages is NF-κB-dependent

**Figure 6:**
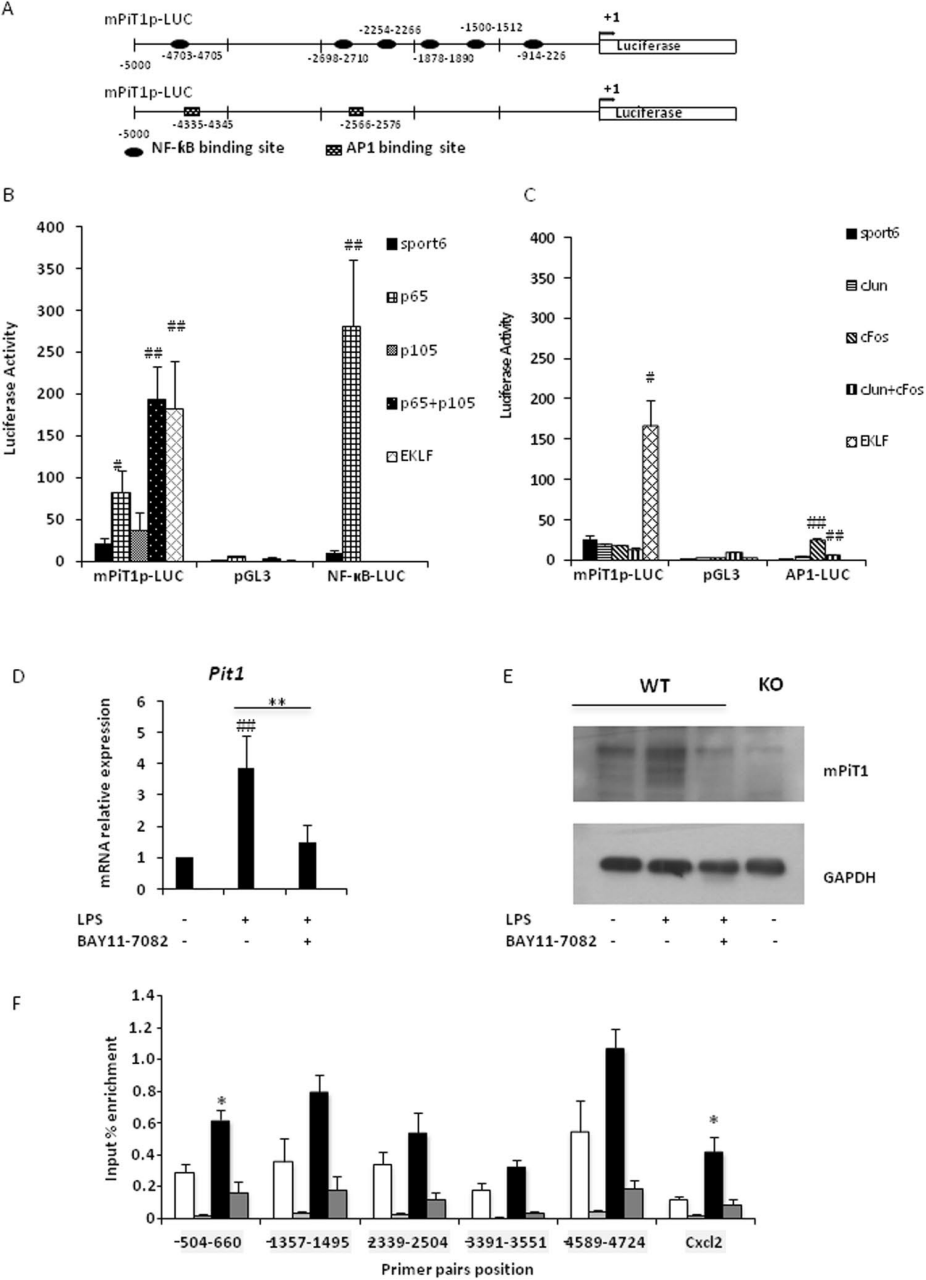
Activity of the mouse *Pit1* promoter is upregulated by p65/ NFκB. (**A**) Schematic representation of m*Pit1*p cloned upstream of the luciferase cDNA in a pGL3-LUC plasmid (mPiT1p-LUC). *In silico* analysis identified six putative binding sites for the p65 subunit of NF-κB (upper panel) and two putative binding sites of AP-1 (lower panel). (**B**) Analysis of *mPit1p* activity by luciferase assay following p65 transfection. HEK293 cells were cotransfected with m*Pit1*-LUC (or pGL3-LUC) and sport6 control plasmid or plasmid encoding p65, p105, or EKLF (positive control). Luciferase activity was normalized to that of the pGL3-LUC, which is devoid of any regulator region upstream of the luciferase cDNA. Data are means ± S.E.M. of at least three experiments. Mann-Whitney Rank Sum test was performed. ^#^p < 0.05; ^##^p < 0.01 vs pGL3-LUC + sport6 condition. (**C**) Analysis of m*Pit1*p activity by luciferase assay following AP-1 transfection. HEK293 cells were cotransfected with m *Pit1*-LUC (or pGL3-LUC) and sport6 control plasmid or plasmid encoding the c-Jun or c-Fos. Luciferase activity was normalized to that of the pGL3-LUC. Data are means ± S.E.M. of at least three experiments. Mann-Whitney Rank Sum test was performed. ^#^p < 0.05; ^##^p < 0.01 vs pGL3-LUC + sport6 condition. (**D**) RT-qPCR analysis of *Pit1* mRNA expression in WT MEFs treated with 20 μM BAY11-7082 or vehicle for 30 minutes prior to stimulation with 100 ng/ml. Data are means ± S.E.M. of at least three experiments. Student’s unpaired t-test or an Unpaired t-test with Welch correction for groups with unequal variance was performed ^#^indicates comparison with the untreated condition: ^##^p < 0.01; *indicates comparison with LPS only treated cells; **p < 0.01. (**E**) Representative western blot of PiT1 in WT MEFs treated with 20 μM BAY11-7082 or vehicle for 30 minutes prior to stimulation with 100 ng/ml LPS for 4 h. Full-length blots are presented in Supplementary Fig. 9. (**F**) q-PCR analysis of p65 ChIP experiments. DNA immunoprecipitated with p65 from MEFs treated (black bar) or not (white bar) with 100 ng/ml LPS for 90 min was analyzed by quantitative PCR using primers located along the mouse *Pit1* promoter. Negative controls (grey bars) were performed using DNA incubated with beads but without anti-p65 antibody. Cxcl2 was used as positive control. Means ± S.E.M. of three experiments are presented. Student’s unpaired t-test or an Unpaired t-test with Welch correction for groups with unequal variance was performed *indicates significant difference from untreated control cells at p < 0.05.

Since *Pit1* expression increased following LPS treatment, we chose to study the regulation of *Pit1* by transcription factors involved in the LPS-TLR pathway. We focused on NF-κB, since *Mcp-1*, *Il-6*, and *Tnfα* are all known NF-κB target genes. The *in silico* analysis of the 5-kilobase mouse *Pit1* promoter (m*Pit1*p) revealed six putative binding sites for NF-κB and two binding sites for AP1 (Fig. 6A). m*Pit1*p was subcloned upstream of the luciferase gene (m*Pit1*p-LUC) as previously described^13^, and the luciferase activity was measured in HEK293 cells transfected with this vector and a plasmid or combination of plasmids encoding p65, p105, c-JUN, or c-FOS. The expression of p65 increased m*Pit1*p activity by more than 4-fold, and the combination of p105 and p65 increased m*Pit1*p activity by 9-fold (Fig. 6B), demonstrating that *Pit1* expression is regulated by the NF-κB pathway. No increase was observed after cotransfection of m*Pit1*p with AP1 (Fig. 6C).

To confirm that *Pit1* regulation is NF-κB dependent, we stimulated MEFs with 100 ng/ml LPS in the presence of a selective pharmacological inhibitor of NF-κB, BAY11-7082. The NF-κB inhibitor blocked upregulation of *Pit1* mRNA (Fig. 6D) and PiT1 protein (Fig. 6E). This confirms that *Pit1* is regulated by NF-κB upon LPS stimulation. Similar results were obtained when BMDMs were treated with 10 ng/ml LPS and BAY11-7085 (Fig. S8). Using a chromatin immunoprecipitation (ChIP) assay, we investigated whether there is a direct interaction of NF-κB with the *Pit1* promoter. ChIP experiments confirmed the direct binding of p65 to the proximal region of the *Pit1* promoter (Fig. 6F).

### PiT1 depletion is associated with impaired NF-κB activation

**Figure 7:**
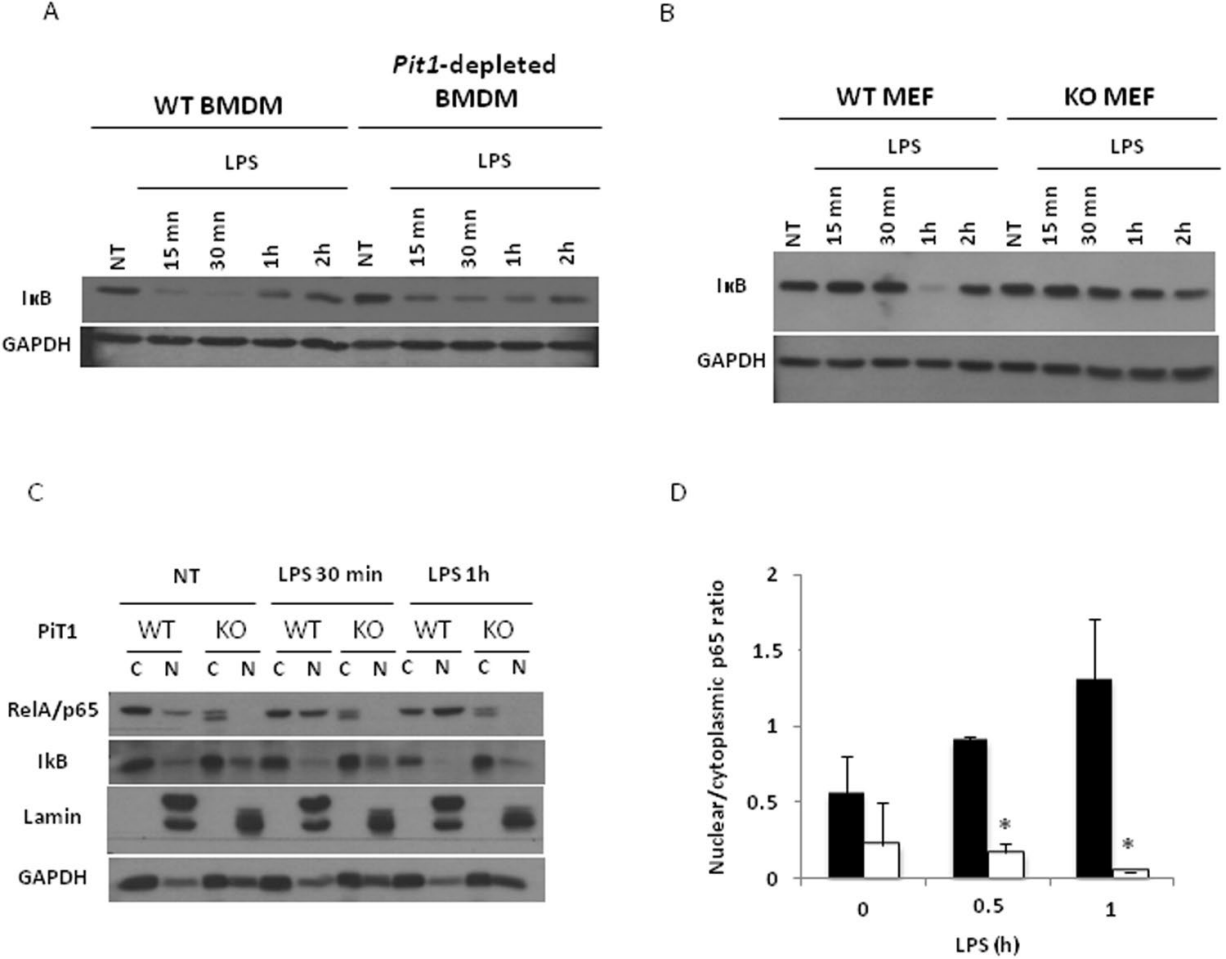
PiT1-depletion is associated with impaired NF-κB activation. (**A**) Representative western blot of IκB degradation in control and PiT1-deficient BMDMs following LPS stimulation. BMDMs from *Mx1-Cre*; *Pit1*^*lox*/*lox*^ and control mice were stimulated *in vitro* with 10 ng/ml LPS for the indicated times (three independent experiments were performed). (**B**) Representative western blot of IκB degradation in WT and *Pit1-*KO MEFs following LPS stimulation (3 independent experiments were performed). MEFs were stimulated *in vitro* with LPS 100 ng/ml for the indicated times. (**C**) Representative western blot of p65 in nuclear (N) and cytoplasmic (C) extracts of WT and *Pit1*-KO MEFs following LPS stimulation. MEFs were stimulated *in vitro* with 100 ng/ml LPS for the indicated times. At least three independent experiments were performed. (**D**) Quantification of nuclear/cytoplasmic p65 ratio in WT and *Pit1*-KO MEFs. Mann-Whitney Rank Sum test was performed and significant differences between WT and *Pit1*-KO MEFs are indicated (*p < 0.05). Full-length blots are presented in Supplementary Fig. 9.

To better assess how PiT1 influences the expression of NF-κB target genes, we next examined the NF-κB pathway following LPS stimulation in control and PiT1-deficient BMDMs as well as in WT and *Pit1*-KO MEFs. Whereas control BMDMs and MEFs displayed the expected degradation of IκBα following LPS treatment, PiT1-deficient BMDMs and MEFs showed an impairment in IκBα degradation (Fig. 7A,B), suggesting that the NF-κB pathway may be less activate in PiT1-deficient cells than in WT cells. Consistent with this result, we found a lower p65 signal in the nuclei of *Pit1*-KO MEFs following LPS stimulation than in WT nuclei (Fig. 7C,D), suggesting that p65 nuclear translocation is impaired in the absence of PiT1.

## Discussion

**Figure 8:**
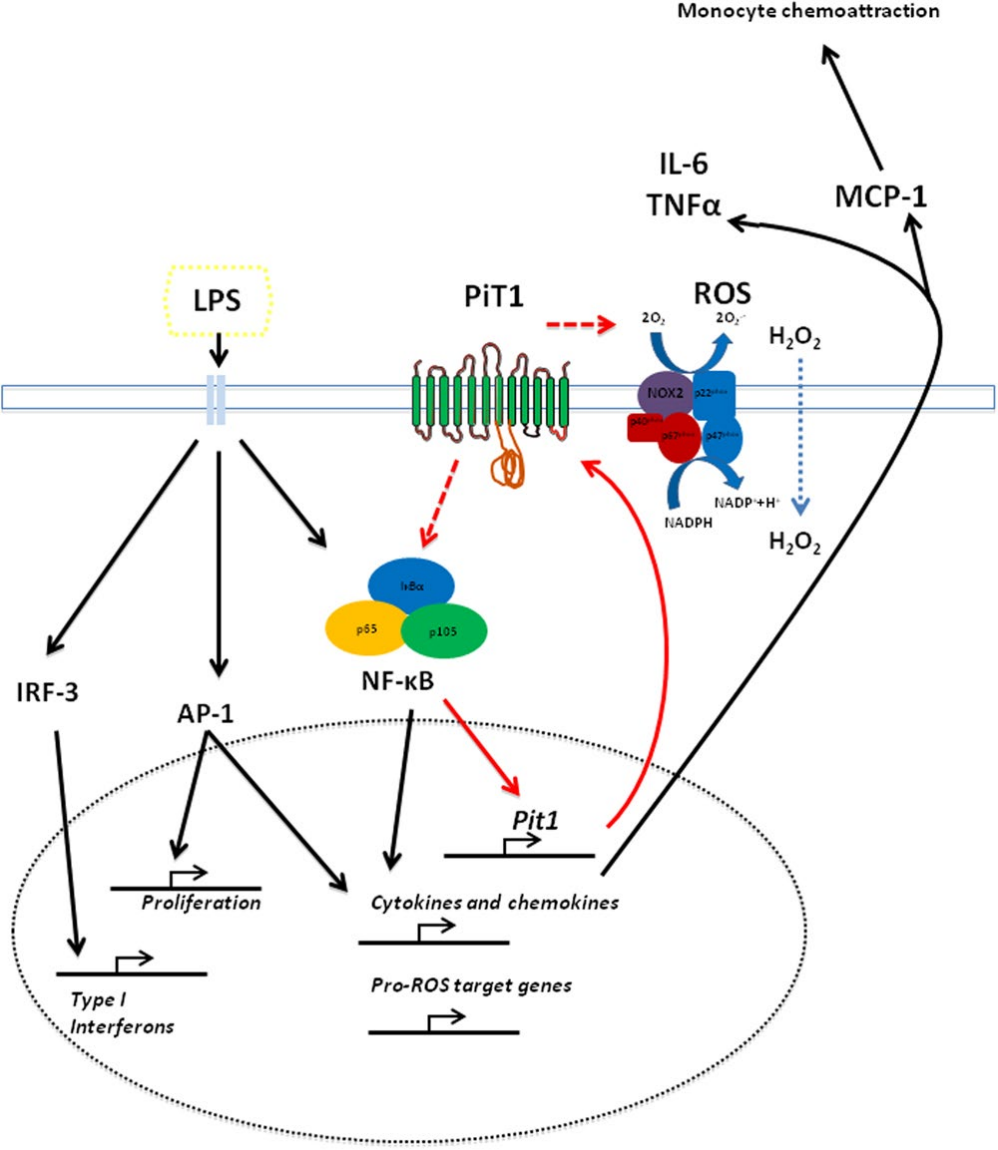
Schematic representation of the function of PiT1 in NF-κB mediated inflammatory response. Toll-like receptor activation by LPS induces the NF-κB, AP1, and IRF3 signaling pathways. NF-κB activates *Pit1* transcription and protein synthesis, as well as synthesis of pro-inflammatory target genes such as *Mcp-1*, *Tnfα*, and *Il-6*. PiT1, in turn, participates in the inflammatory response by promoting NF-κB activation.

In the present work, and as summarized in Fig. 8, we showed that chemokine *Mcp-1* mRNA levels and MCP-1 concentration were impaired *in vitro* in BMDMs depleted of PiT1 (also known as Slc20a1) and in *Pit1-*KO MEFs upon LPS stimulation and *in vivo* in the LPS-induced inflammation model in *Mx1-Cre*; *Pit1*^lox/lox^ mice compared to control mice. Furthermore, major functions such as ROS production and wound healing were impaired in PiT1-deficient BMDMs. Notably, reduced IκB degradation and lower p65 nuclear translocation were observed in *Pit1*-KO MEFs upon LPS stimulation, suggesting an impact of PiT1 depletion on NF-κB pathway activation. We found that LPS induces the transcription of *Pit1* in an NF-κB-dependent manner. Indeed, the physiological activation of the NF-κB pathway by LPS triggers PiT1 expression. Additionally, the disruption of the NF-κB pathway with pharmacological inhibitors of NF-κB abolishes *Pit1* upregulation. Importantly, our study provides the first experimental evidence that p65/NF-κB is able to transactivate the *Pit1* promoter: LPS treatment resulted in the recruitment of endogenous p65 to the *Pit1* promoter.

Little is known about the regulation of *Pit1* transcription. Our group has previously demonstrated that *Pit1* is a target gene of EKLF, a transcription factor involved in erythroid differentiation^13^. Although global surveys (microarray and computer-based analysis) identified hundreds of potential NF-κB-responsive genes^41,42^, listed in the following websites: http://www.bu.edu/nf-kb/the-gilmore-lab/ and http://bioinfo.lifl.fr/NF-KB. *Pit1* is not among these. The upregulation of *Pit1* mRNA in response to diverse treatments known to activate the NF-κB pathway, including TNFα (of pre-adipocytes^22^), IL1α or PMA (of hematopoietic cell lines^23^), IGF1 (of immortalized fibroblasts^43^), and TGFβ or oxidized low density lipoproteins (of chondrocytes^44^) has been previously described. Interestingly, *PiT1*, but not *PiT2*, was identified as a possible NF-κB target gene in the liver after VHL inactivation^24^ and is present in the list of genes induced by TNFα^22^, suggesting a PiT1-specific induction of NF-κB mediated inflammation. In the present study, we did not observe an effect of LPS on *Pit2* expression. The higher expression of *Pit2* that we observed in PiT1-deficient BMDMs following LPS stimulation suggests a compensatory increase in *Pit2*, which does not, however, prevent the effects of PiT1 depletion on MCP-1 and IL-6 levels. Further experiments will be necessary to determine whether these effects are Pi-transport dependent or not.

We also provide evidence showing that PiT1 is involved in the LPS response *in vivo*, as demonstrated by the lower amounts of IL-6 and MCP-1 in the serum of *Mx1-Cre*; *Pit1*^*lox*/*lox*^ mice after LPS stimulation, which suggests that PiT1 may play a role in innate immunity. Proinflammatory cytokines and chemokines are known NF-κB target genes (http://www.bu.edu/nf-kb/the-gilmore-lab/) and are involved in a wide variety of pathological and inflammatory conditions^33^ including rheumatoid arthritis, multiple sclerosis, and glomerulonephritis^45–47^.

Our results also provide evidence showing that PiT1-deficient macrophages have reduced ROS production. High levels of ROS can lead to cellular damage, oxidative stress, and DNA damage. In phagocytic cells such as macrophages, ROS production, which is necessary for bactericidal action, is mainly catalyzed by the action of the NADPH oxidase NOX2, a membrane-bound enzyme complex^48^. The NF-κB pathway is a major regulator of ROS production; it fine-tunes the expression of anti-oxidant and pro-ROS genes^49^. ROS also influence the NF-κB pathway^48^. As both PiT1 and NOX2 are expressed at the cell surface, PiT1 and the NOX complex may interact directly. It is also possible that a decrease in *NOX2* gene transcription regulated by NF-κB, or expression of NF-κB pro-inflammatory target genes such as *Tnfα*, which enhance ROS production in an autocrine manner^50^, could explain at least in part the reduced ROS observed in PiT1-deficient cells in the present work.

Importantly, PiT1 was identified as a direct positive modulator of the NF-κB pathway in a global screen^51^. In this study, we found a reduced nuclear translocation of p65 in PiT1-deficient cells, which is consistent with the altered degradation of IκBα in these cells. Although the exact mechanism has not been elucidated, this result suggests that PiT1 interferes with the activation of NF-κB pathway following LPS treatment.

Many of the signaling events leading to cytokine synthesis and release following LPS exposure are now well established^52–55^. The transcription factor NF-κB is critical for the expression of these inflammatory proteins and is regarded as the master regulator of the immune response^56^. The transcriptional activity of NF-κB is primarily regulated through its sequestration in the cytoplasm by the IκB family proteins^57^. Upon stimulation by a toll-like receptor ligand or TNFα, IκBα is phosphorylated by the IKK complex, which targets it for ubiquitination and subsequent proteasome-mediated degradation. NF-κB dimers then translocate into the nucleus and bind to regulatory regions of target genes^25^. The ubiquitination and proteasomal degradation of NF-κB is critical in the termination of the NF-κB transcriptional response and represents a major limiting factor in the expression of proinflammatory genes^58–60^. Ubiquitination and deubiquitination processes are also required at other steps of the NF-κB pathway. Indeed, LPS-induced activation of NF-κB is known to require the activating K63 and M1 ubiquitination of NEMO and TRAFs proteins^25^.

Interestingly, a yeast-two hybrid screen performed by our group identified putative interactions of PiT1 with several ubiquitinating and deubiquitinating enzymes such as ligases UBC9 and SIAH2 and protease USP7. USP7 proteolytically removes polyubiquitin chains from substrates^61^. Among the identified targets of USP7 are tumor suppressor protein P53^62^, its regulator MDM2^63^, and PTEN^64^. Importantly, NF-κB p65 was recently identified as a substrate for USP7 deubiquitinase activity in the nucleus, where USP7 is recruited to NF-κB target promoters and interacts with NF-κB in a DNA-binding-dependent manner^65^. USP7 deubiquitination of NF-κB leads to increased transcriptional activity and expression of target genes in response to TLR and TNFα-receptor activation. In a recent work, we confirmed that USP7 binds to PiT1 and demonstrated that *Pit1* deletion inhibited USP7/IRS1 dissociation upon insulin stimulation^15^. This prevented IRS1 ubiquitination and its subsequent proteasomal degradation. Among the other suggested partners of USP7 is RIP1^66^. RIP1 is known to be involved in the LPS-TLR4 pathway, and its ubiquitination is a crucial checkpoint that determines whether NF-κB-mediated transcription is activated. TRIF adaptor protein, indirectly via TRAM, binds to RIP1 and TRAF6 following receptor activation, which can lead to the activation of NF-κB- and MAPK-mediated signaling^67^. Further work will be needed to decipher the molecular mechanism underlying the impact of PiT1 on the NF-κB pathway and possible interactions with ubiquitinating and deubiquitinating enzymes.

Our study does have limitations. Our previous research showed that, in an allelic series of mutant mice, a phenotype was observed only when *Pit1* expression was reduced to below 15% of WT levels^4^. Moreover our *in vitro* studies using siRNA-mediated gene silencing revealed that PiT1-depletion must be very effective to observe phenotype (data not shown). For this reason, we decided to use MEFs or BMDM *Pit1*-knockout cells instead of cells in which *Pit1* was depleted using siRNA. *Pit1* gene expression was checked in each experiment involving the *Mx1-Cre* model. BMDMs expressing *Pit1* more than 5% of the controls were excluded. Furthermore, with the *Mx1-Cre* system, *Pit1* is deleted not only in macrophages but also in other organs. This may explain some discrepancies between *in vitro* and *in vivo* data. In particular, we observed higher IL-10 serum level in *Mx1-Cre*; *Pit1*^*lox*/*lox*^ mice compared to controls, whereas IL-10 was found to be lower *in vitro* in PiT1-deficient BMDMs. This correlates with the phenotype of *Mx1-Cre*; *Pit1*^*lox*/*lox*^ mice characterized by liver inflammation (color change and increased amino acid transferases, data not shown), which suggests that IL-10 is increased in this *in vivo* model to dampen excessive inflammation. Therefore it is difficult to draw conclusions on IL-10 and to attribute all the *in vivo* results to PiT1 depletion in macrophages only. Although the *Mx1-Cre* system is active in numerous organs, we observed consistently lower *Mcp-1* mRNA and MCP-1 protein levels in PiT1-deficient macrophages, as well as lower MCP-1 in the serum of *Mx1-Cre*; *Pit1*^*lox*/*lox*^ mice. This suggests that the *in vivo* effects are likely due in part to PiT1 depletion in macrophages.

In summary, we have uncovered a previously unsuspected role for PiT1 in the LPS-mediated inflammatory response and provide mechanistic insights into the regulation of PiT1 expression by NF-κB. We showed that LPS-induced *Pit1* upregulation is NF-κB dependent. Thus, PiT1 may enhance the production of pro-inflammatory and chemoattractant mediators and ROS. In future studies, it will be interesting to investigate the physiological impact of NF-κB-dependent PiT1 up-regulation in pathophysiological contexts.

## Materials and Methods

### Animals

The mice used in the study were on a 129sv/J × C57BL/6J mixed background. Animal care and maintenance were provided through the University Paris Descartes accredited Animal Facility at Necker Faculty of Medicine (Paris). Mice were maintained on rodent laboratory chow (Special Diet Services). All procedures were approved by the Animal Care and Use Committee of the University Paris Descartes No. 034 and the Ministère de l’Enseignement Supérieur et de la Recherche (Laboratoire d’Expérimentation Animale et Transgénèse (LEAT) IFR agreement number A751408) and were performed in accordance with the 2010/63/UE directive. *Pit1* genetically modified mice were generated as described in^4^. *Pit1*^*lox*/+^ animals were intercrossed to *Pit1*^*lox*/*lox*^ mice. *Pit1*^*lox*/*lox*^ ^4^ mice were then crossed with *Mx1-Cre* mice^68^, provided by Dr. Thomas Mercher (INSERM U985, Université Paris XI, Villejuif, France) to obtain the conditional *Pit1* strain *Mx1-Cre*; *Pit1*^*lox*/*lox*^. *Mx1-Cre*; *Pit1*^*lox*/*lox*^ mice were then bred with *Pit1*^*lox*/*lox*^ mice in order to obtain controls (*Pit1*^*lox*/*lox*^) and experimental animals (*Mx1-Cre*; *Pit1*^*lox*/*lox*^) from the same litters. The presence of the *Mx1-Cre* transgene was confirmed by PCR using the following primers: Forward 5′-AGCCTGGGGGTAACTAAACTGG-3′, Reverse 5′-CCATTGCCCCTGTTTCACTATC-3′. Tissue-specific deletion of *Pit1* was induced by three intraperitoneal injections of polyinosinicpolycytidylic acid (pIpC, #tlrl-picw, Invivogen) at 2-day intervals at 5 weeks of age^68^, and mice were analyzed at least 6 weeks after injection to avoid acute side effects due to IFN production. Control mice were also injected with pIpC to normalize for potential sustained effects resulting from pIpC treatment.

### Cells and cell culture conditions

For the generation of BMDMs, femurs and tibiae of 12 to 16-week-old mice were flushed with RPMI-1640 medium (Gibco, 61870–010). BMDMs were selected by adhesion to petri dishes after 7 days of differentiation in RPMI-1640 supplemented with 10% fetal bovine serum (FBS) and 20% M-CSF-containing medium^69^. M-CSF-containing medium was obtained by harvesting conditioned medium from L929 mouse fibroblast cells at confluency and at 7 days past confluency. For any further experimental procedures, cells were detached, counted, seeded and cultivated in RPMI-1640 with 10% FBS without L929-conditioned medium. For ROS procedures, RPMI-1640 without phenol red (Gibco, 11835) was used.

MEFs were isolated as previously described^4^ and were maintained in DMEM (Gibco 41965–039) supplemented with 10% FBS. Cells were cultured at 37 °C in 5% CO_2_ in a humidified atmosphere. HEK293 cells were grown in DMEM/F12 (Gibco, 31330–038) supplemented with 10% FBS at 37 °C in 5% CO_2_.

Cells were stimulated with complete medium containing lipopolysaccharide (LPS) from *E*. *coli* strain O111:B4 (Invivogen, #tlrl-3pelps) at a concentration of 10 ng/ml for BMDMs or 100 ng/ml for MEFs as described previously^31,70^ and following preliminary tests suggesting that a 10 ng/ml dose in BMDMs and a 100 ng/ml dose in MEFs were the optimal concentrations to activate the NF-κB pathway (assessed by IκB degradation) and to induce pro-inflammatory cytokine expression in these two different cell types. In experiments using the NF-κB inhibitor BAY11-7082 (Calbiochem, 196870) and BAY11-7085 (Selleckchem, S7352), cells were incubated for 30 min in the presence of 20 μM of the drug or vehicle before stimulation with LPS 10 or 100 ng/ml.

### Isolation of macrophages from the peritoneal exudate

To induce inflammatory exudates, male *Mx1-Cre*; *Pit1*^*lox*/*lox*^ and control mice were injected intraperitoneally at 12–16 weeks of age with 1 mL of 4% brewer thioglycollate medium (Sigma, B2551) or the same volume of PBS. These mice were sacrificed 72 h later, and the peritoneal exudate cells were obtained by injecting 5 mL of ice-cold 0.5% BSA in PBS into the peritoneal cavity. After a gentle massage, the fluid was harvested, and the cells were centrifuged and washed. Peritoneal cells were FACS-phenotyped as described below.

### Flow cytometry analysis

BMDMs and peritoneal macrophages were incubated with the fluorescent dye-conjugated antibodies for 30 min at 4 °C, briefly washed in 0.5% BSA in PBS and analyzed using FORTESSA (Beckton Dickinson) after DAPI (Molecular Probes, D3571) or Sytox blue (Molecular Probes, S34847) staining to allow exclusion of dead cells. The antibodies used were: FITC-conjugated rat anti-mouse CD11b (BD Biosciences, 553310), rat anti-mouse F4/80 (Abcam, ab6640) with Alexa Fluor 594 conjugated donkey anti-rat IgG (H + L) secondary antibody (Thermo Fisher, A-21209), and the relevant isotype controls. Ten thousand total events were collected and gated for analysis of live cells.

### Macrophage phagocytosis assay

Macrophage phagocytosis of *E*. *coli* substrate was assessed using the CytoSelect 96-well phagocytosis assay kit (Cell Biolabs, CBA 222). Briefly, 50,000 BMDMs were seeded in each well of a 96-well plate and incubated overnight at 37 °C in 5% CO_2_. Phagocytes were treated with 10 ng/ml LPS for 30 minutes before adding 10 μL of *E*. *coli* suspension. The number of engulfed particles was determined 6 h later as described in the manufacturer’s protocol. Each sample was assayed in duplicate. Two independent experiments were performed. BMDMs from three male *Mx1-Cre*; *Pit1*^*lox*/*lox*^ and three control mice were analyzed in each experiment. Absorbance measurements were performed on a microplate autoreader (Berthold, Mithras) with a 450-nm filter.

### Cytokine detection

Supernatants from BMDMs and MEFs cultures were collected after incubation with LPS for the indicated times and then analyzed for MCP-1, IL-6, and TNFα. Cytokine levels were determined by quantitative sandwich immunoassay technique (Quantikine, R&D, MJE00, M6000B, MTA00B assays, respectively) according to manufacturer’s instructions. Concentrations of serum cytokines from control and *Mx1-Cre*; *Pit1*^*lox*/*lox*^ mice were determined using a Biorad Mouse Cytokine multiplex Assay.

### *In vitro* wound-healing assay

BMDMs from *Mx1-Cre*; *Pit1*^*lox*/*lox*^ and control mice were seeded (1.8 × 10^6^ cells/ml) on IBIDI µ-Slide 8-well glass bottom plates with culture inserts (IBIDI, 80209) in RPMI-1640 with 10% FBS. After 72 h, IBIDI inserts were removed and closure of the resulting wound was photographed every 24 h over the next 72 h. Images of the wounds were captured using a Zeiss Observer Z1 microscope with a 10x (NA/0.5) objective. Images were analyzed with ImageJ software.

### Measurement of intracellular ROS

ROS production was determined using CM-H2DCFDA (Molecular Probes, C6827), which is de-esterified upon entering cells and then oxidized to fluorescent DCF by intracellular ROS. After 7 days of differentiation, BMDMs from *Mx1-Cre*; *Pit1*^*lox*/*lox*^ and control mice were seeded in 6-well plates at 1 × 10^6^ cells/well and were incubated in phenol red-free RPMI 1640 medium containing 10% FBS for 6 h. The cells were then incubated in the presence of 1000 ng/ml LPS or vehicle for 20 h. The cells were detached and incubated in PBS with CM-H2DCFDA at 10 μM for 45 min. Cell viability was assessed using DAPI staining. Living cells, which are DAPI negative, were selected by FACS gating. In these living cells, we recorded the fluorescence of DCF by flow cytometry (excitation 488 nm) on a Fortessa instrument (Beckton Dickinson). Positive controls were treated with H_2_O_2_ (Sigma, H1009) at a final concentration of 500 μM for 1 h. BMDMs pooled from at least five control or five *Mx1-Cre*; *Pit1*^*lox*/*lox*^ mice were analyzed in three independent experiments. Data were processed and normalized to values obtained from untreated controls.

### Measurement of ROS production by luminol-amplified chemiluminescence

ROS production was measured by the luminol-amplified chemiluminescence method. Briefly, BMDMs from *Mx1-Cre*; *Pit1*^*lox*/*lox*^ and control mice (2.5 × 10^5^ cells) isolated as described above were resuspended in 500 µl of HBSS and pre-incubated 10 min at 37 °C in the presence of 10 µM luminol and 5 U HRP then stimulated with fMLF at a final concentration of 10^−5^ M. Chemiluminescence was measured using a luminometer (Biolumat LB937; Berthold), which converts light intensity into counts per minute (cpm).

### Luciferase assay

The 5-kbp of the mouse *Pit1* promoter (m*Pit1*p) was cloned upstream of the *luciferase* gene (*LUC*) in a pGL3-LUC plasmid (m*Pit1*p-LUC) as previously described^13^. HEK293 cells were plated in 24-well plates prior to transient transfection with Lipofectamine 2000 reagent (Invitrogen, 11668–019). Cells were cotransfected with 1 μg of m*Pit1*p-LUC construct and 500 ng of transcription factor expression plasmid. To correct for transfection efficiency, all cells were cotransfected with 100 ng of the pRL-tk plasmid (Promega, E2241), expressing the *Renilla reniformis* luciferase. The assay was performed as described previously^13^. The cDNAs encoding p65 and p105 were purchased from Biovalley and subcloned into a pSPORT6 expression plasmid (pSPORT6-p65, pSPORT6-p105).

Negative controls were performed by cotransfecting cells either with the m*Pit1*p-LUC construct and an empty expression vector or with an empty pGL3-LUC plasmid and a transcription factor expression vector. An artificial construct containing three NF-κB sites upstream of *LUC* was used as positive control (NF-κB-LUC) for NF-κB activation. The transcription factor EKLF, recently shown by our group to activate the *Pit1* promoter^13^, was used as positive control for m*Pit1*p-LUC activity. Activities of firefly and *Renilla* luciferases were analyzed 48 h after transfection using the Dual-Luciferase Reporter Assay System (Promega, E1910) according to the manufacturer’s instructions. The firefly luciferase activity was normalized to *Renilla* luciferase expression. Three to five independent experiments were performed.

### Gene expression analysis

Total RNA was isolated from cells using Nucleospin RNA II columns (Macherey Nagel, 740955.250). RT-PCR amplifications were performed using M-MLV (Invitrogen, 28025–013) according to manufacturer’s instructions. Q-PCR was performed using SyBr Green chemistry (Thermo Fischer Scientific). The *Pinin* gene was used as the reference gene, and levels were calculated using the 2^−ΔΔCT^ method and expressed relative to the mean value of untreated samples^71^.

### Western blotting

Cells were incubated for 30 min in ice-cold lysis buffer (150 mM NaCl, 10 mM Tris HCl, 5 mM EDTA, 1% NP-40, 0.1% SDS, 0.5% deoxycholate, 1 mM Na_3_VO_4_, 1 mM NaF, 5 mM sodium pyrophosphate, and a protease inhibitor cocktail). After centrifugation at 20,000 g for 15 min, supernatants were boiled in 1xSDS loading buffer prior to SDS-PAGE. Proteins were transferred to PVDF membrane and blocked with 5% milk/TBST (10 mM Tris, pH 7.5, 150 mM NaCl, 0.15% Tween 20) for 1 h. Subcellular fractionations were performed using NE-PER nuclear and cytoplasmic extraction reagents (Thermo Scientific, P178833) according to manufacturer’s instructions. Blots were probed with primary antibodies in 5% BSA overnight at 4 °C followed by HRP-conjugated secondary antibodies if required and subsequent ECL detection reagent (Thermo Fischer, Pierce ECL Western Blotting Substrate 32106). The PiT1 antibody was prepared in our lab and is an anti-mouse polyclonal rabbit antibody^9^. Antibodies against IκB (Cell Signaling, 4814), p65 (Cell Signaling, 4764), lamin (Cell Signaling, 2032), and GAPDH (Santa Cruz Biotechnology, sc-25778) were purchased.

### Chromatin immunoprecipitation assay

ChIP assays were performed as described^13^. In brief, the chromatin was fragmented into approximately 500- to 1000-bp fragments by sonication (5 × 1 s, with 0.5 s intervals) at intensity 30% (Branson Digital Sonifier), and then 20 μg of sonicated DNA was incubated overnight at 4 °C with 2 μl of anti-p65 antibody (Cell Signaling, CS8242). The control for non-specific DNA immunoprecipitation was produced by amplifying a fragment of the *β-actin* gene. The DNA-containing supernatant was analyzed by qPCR. Primer pairs for amplification of the mouse *Pit1* promoter region containing NF-κB putative binding sites are detailed in Table 1. Relative occupancy values were calculated by determining the apparent immunoprecipitate efficiency as the ratio of the amount of immunoprecipitated DNA over that of the input sample.

**Table 1:** PCR primers.

Mouse *Pit1* promoter	5′-3′ sequence
Forward-660	TACATGGGGAAAGGGAAAGGAC
Reverse-504	GGGGACATGGGAACACTCACT
Forward-1495	AGGGGGAAAGAAGGAGGAAGT
Reverse-1357	ACAGAGAAGGAAGCGGCGTT
Forward-2504	CCGAATTAGTGAACGATGGACA
Reverse-2339	CCTCTCCAACCTCACCCTCAG
Forward-3551	ATGGAAGGTGGAGGGAGCGT
Reverse-3391	GTAGTTCCCAAGGTCGGTCTG
Forward-4724	TGCCAGGCCAGGACAAGT
Reverse-4589	CCTCCAGCCGCACTTTCAG

### Statistical analyses

Results are presented as means ± S.E.M. For statistical analyses, significance was tested using the Student’s unpaired t-test or an Unpaired t-test with Welch correction for groups with unequal variance, or the Mann-Whitney Rank Sum test when data did not follow a normal distribution. The ANOVA test and Tukey’s multiple comparison test were performed for time-courses. In all cases, the level of statistical significance was set at p < 0.05.

## Supplementary information

Supplementary figures

## Data Availability

All data generated or analyzed during this study are included in this published article (and its Supplementary Information Files) or are available from the corresponding author on reasonable request.
