# Supplementary material for: Novel function of PiT1/SLC20A1 in LPS-related inflammation and wound healing
Source: Sci Rep. 2019 Feb 12;9:1808. doi: 10.1038/s41598-018-37551-1 (PMC6372663; doi:10.1038/s41598-018-37551-1)

Novel function of PiT1/SCL20A1 in LPS-related inflammation and wound healing

Eugénie Koumakis1 2 3*, Joëlle Millet-Botti 1 4, Jamel El Benna5, Christine Leroy1, Valérie Boitez1, Patrice Codogno1, Gérard Friedlander#1, Anne Forand#6

#These authors contributed equally to this study.

1 INSERM UMR_S1151 CNRS UMR8253 Institut Necker-Enfants Malades (INEM) Université Paris Descartes
2 Rheumatology Department A, Cochin Hospital, APHP, Paris
3 Centre de Référence des Maladies Rares du métabolisme du Calcium et du Phosphate, site constitutif, Cochin Hospital
4 Université Paris Diderot-Sorbonne Paris Cité, F-75993 Paris, France
5 INSERM U1149, CNRS-ERL8252, Centre de Recherche sur l'Inflammation, Université Paris Diderot, Sorbonne Paris Cité, Laboratoire d'Excellence Inflamex, Faculté de Médecine, Site Xavier Bichat, 75018 Paris, France
6 Inovarion, Paris

Supplemental Data legends

Figure S1. (A-C) Fold increase in A) *Mcp-1*, B) *Il-6*, and C) *Tnfα* mRNA in BMDMs from *Mx1-Cre;Pit1*$^{lox/lox}$ (white bars) and control mice (black bars) stimulated *in vitro* with 10 ng/ml LPS. **(D-F)** Fold increase in D) MCP-1, E) IL-6, and F) TNFα concentrations in the supernatants of BMDMs from *Mx1-Cre;Pit1*$^{lox/lox}$ (white bars) and control mice (black bars) stimulated *in vitro* with 10 ng/ml LPS. **(G)** Fold increase in *Mcp-1* mRNA in *Pit1*-WT (black bars) and *Pit1*-KO (white bars) MEFs stimulated *in vitro* with 100 ng/ml LPS. (H) Fold increase in MCP-1 concentration in the supernatants of *Pit1*-WT (black bars) and *Pit1*-KO (white bars) MEFs stimulated *in vitro* with 100 ng/ml LPS. Data are means ± S.E.M. of at least three independent experiments. Student's unpaired t-test or an Unpaired t-test with Welch correction for groups with unequal variance was performed. * is comparison between control and PiT1-deficient cells. * $p<0.05$; ** $p<0.01$; *** $p<0.001$(comparisons with untreated conditions are shown on Figure 1).

Figure S2. RT-qPCR analysis of *Tlr4* mRNA expression in non-stimulated BMDMs from *Mx1-Cre;Pit1*$^{lox/lox}$ (white bars) and control mice (black bars). Data were normalized to data from non-stimulated control cells. Data are means ± S.E.M. of three independent experiments. Student's unpaired t-test or an Unpaired t-test with Welch correction for groups with unequal variance was performed.

Figure S3. IL-6 and TNFα levels in *Pit1*-KO and WT MEFs (A-B) RT-qPCR analysis of A) *Il-6* and B) *Tnfα* mRNA expression in *Pit1*-WT (black bars) and *Pit1*-KO (white bars) MEFs stimulated *in vitro* with 100 ng/ml LPS for the indicated time. **(C-D)** ELISA quantification of C) IL-6 and D) TNFα concentrations in supernatants from MEFs stimulated *in vitro* with 100 ng/ml LPS for the indicated time. Data were normalized to data from non-stimulated control cells. Data are means ± S.E.M. of at least three independent experiments. Student's unpaired t-test or an Unpaired t-test with Welch correction for groups with unequal variance was performed; # is comparison with the untreated condition; # $p<0.05$; ## $p<0.01$; ### $p <0.001$.

Figure S4. M2-gene expression in BMDMs. RT-qPCR analysis of *Cd206* and *Il-10* mRNA expression in BMDMs from *Mx1-Cre;Pit1*$^{lox/lox}$ (white bars) and control mice (black bars) stimulated *in vitro* with 10 ng/ml LPS for the indicated times. Data were normalized to those from non-stimulated control cells. Data are means ± S.E.M. of at least three independent experiments. Student's unpaired t-test or an Unpaired t-test with Welch correction for groups with unequal variance was performed; # is comparison with the untreated condition or other time points; * is comparison between control and PiT1-deficient cells. * $p<0.05$; # $p<0.05$; ## $p<0.01$; ### $p<0.001$.

Figure S5. Flow cytometric analysis of macrophages after thioglycollate-induced peritonitis. *Mx1-Cre;Pit1$^{lox/lox}$* (light grey bars) and control (dark grey bars) mice were intraperitoneally injected with 4% thioglycollate or PBS (white bars for transgenic and black bars for control mice) as described in the Materials and Methods section. Three days later, peritoneal exudates were harvested and the total cell numbers were counted and analyzed for F4/80 (Alexa 594) and Cd11b (FITC) markers identifying F4/80^{high} and Cd11b^{high} (resident) and F4/80^{high} and Cd11b^{low} (recruited) macrophages. Data are means ± S.E.M. of at least three independent experiments. Student's unpaired t-test or an Unpaired t-test with Welch correction for groups with unequal variance was performed; # Comparison with the untreated condition; # $p<0.05$.

Figure S6. Phagocytic capacity of BMDMs. Following 7-day differentiation, macrophages were seeded at 50,000 cells per well in 96-well plates. After 24 h, cells were pretreated with 10 ng/ml LPS for 30 min and then incubated in the presence of *E. coli* particles or medium. Absorbance measurements were performed with a 450-nm filter. Data are means ± S.E.M. of two independent experiments. Student's unpaired t-test or an Unpaired t-test with Welch correction for groups with unequal variance was performed; # is comparison with the untreated condition; # $p<0.0001$.

Figure S7. RT-qPCR analysis of *Pit2* mRNA expression. Control (black bars) and PiT1-deficient (white bars) BMDMs were stimulated *in vitro* with 10 ng/ml LPS for the indicated times. Data were normalized to those from non-stimulated control cells. Data are means ± S.E.M. of at least three independent experiments. Student's unpaired t-test or an Unpaired t-test with Welch correction for groups with unequal variance was performed; * indicates comparison between control and PiT1-deficient cells ; $p<0.05$.

Figure S8. Inhibition of NF-κB blocks upregulation of *Pit1* mRNA. RT-qPCR analysis of *Pit1*, *Il-6*, *and Mcp-1* mRNA expression in BMDMs treated with 20 μM BAY11-7085, 20 μM BAY11-7082, or vehicle for 30 min prior to stimulation with 10 ng/ml LPS for 2 h. Data were normalized to those from non-stimulated control cells. Data are means ± S.E.M. of at least three independent experiments. Student's unpaired t-test or an Unpaired t-test with Welch correction for groups with unequal variance was performed; # indicates comparison with the untreated condition; * indicates comparison with LPS only treated cells; * $p<0.05$, ** $p<0.01$; *** $p<0.001$; # $p<0.05$; ## $p<0.01$; ### $p<0.001$.

Figure S9. Uncropped images of western blots shown in Fig. 5B, 5E, 6E, 7A, 7B, 7C.

Figure S1

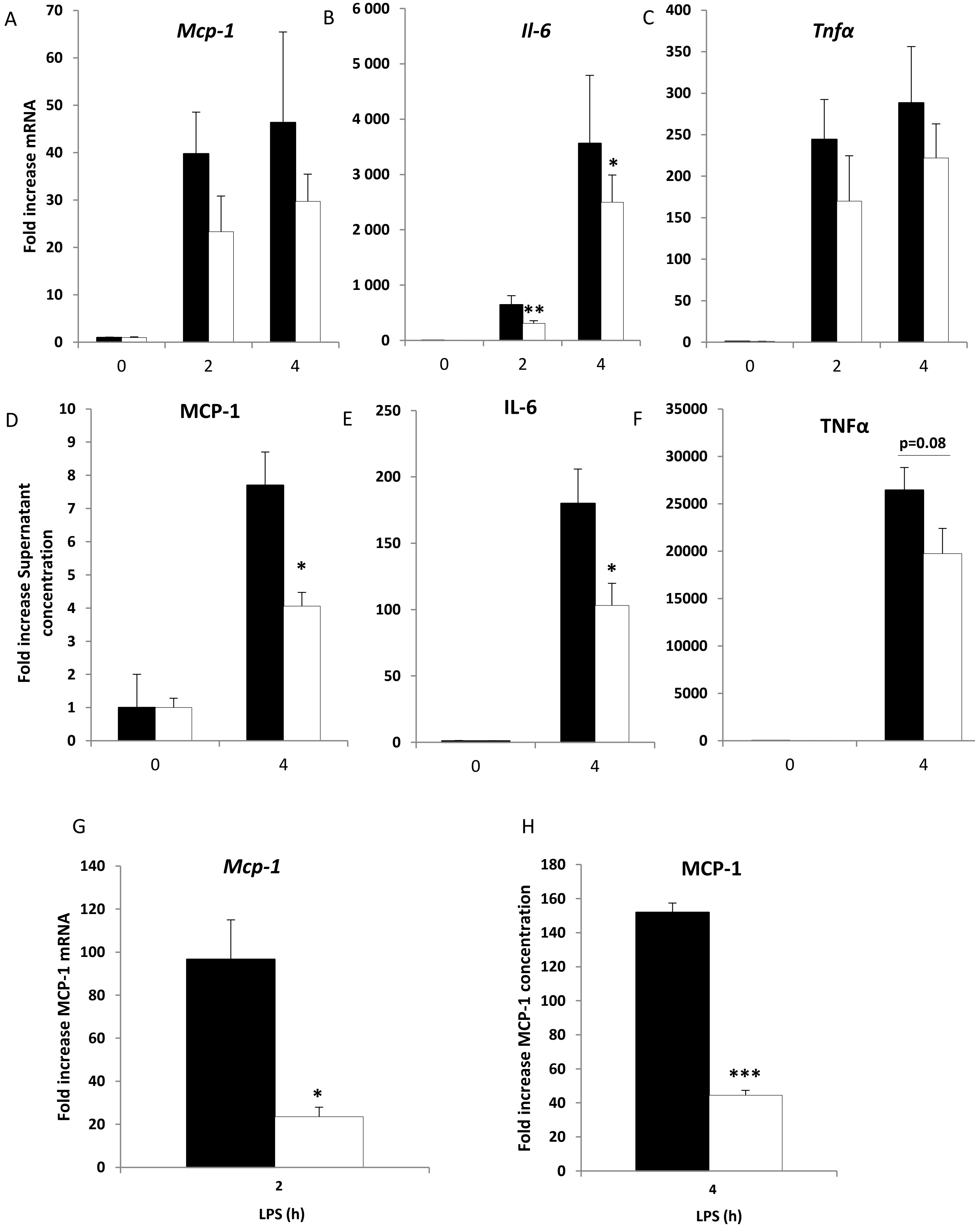
A
Mcp-1
Fold increase mRNA
0
10
20
30
40
50
60
70
0
2
4
B
Il-6
0
1 000
2 000
3 000
4 000
5 000
6 000
**
*
C
Tnfα
0
50
100
150
200
250
300
350
400
D
MCP-1
Fold increase Supernatant concentration
*
E
IL-6
0
50
100
150
200
250
*
F
TNFα
p=0.08
0
5000
10000
15000
20000
25000
30000
35000
G
Mcp-1
Fold increase MCP-1 mRNA
0
20
40
60
80
100
120
140
*
2
LPS (h)
H
MCP-1
Fold increase MCP-1 concentration
0
20
40
60
80
100
120
140
160
180
***
4
LPS (h)

Figure S2

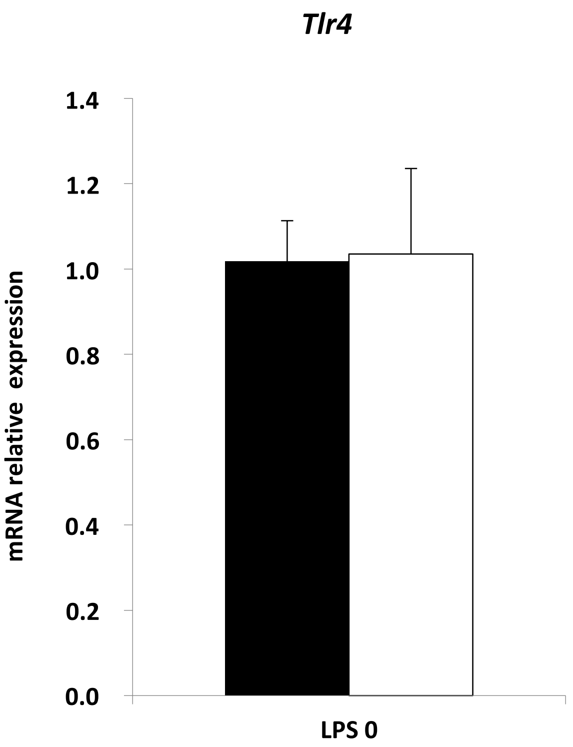

Figure S3

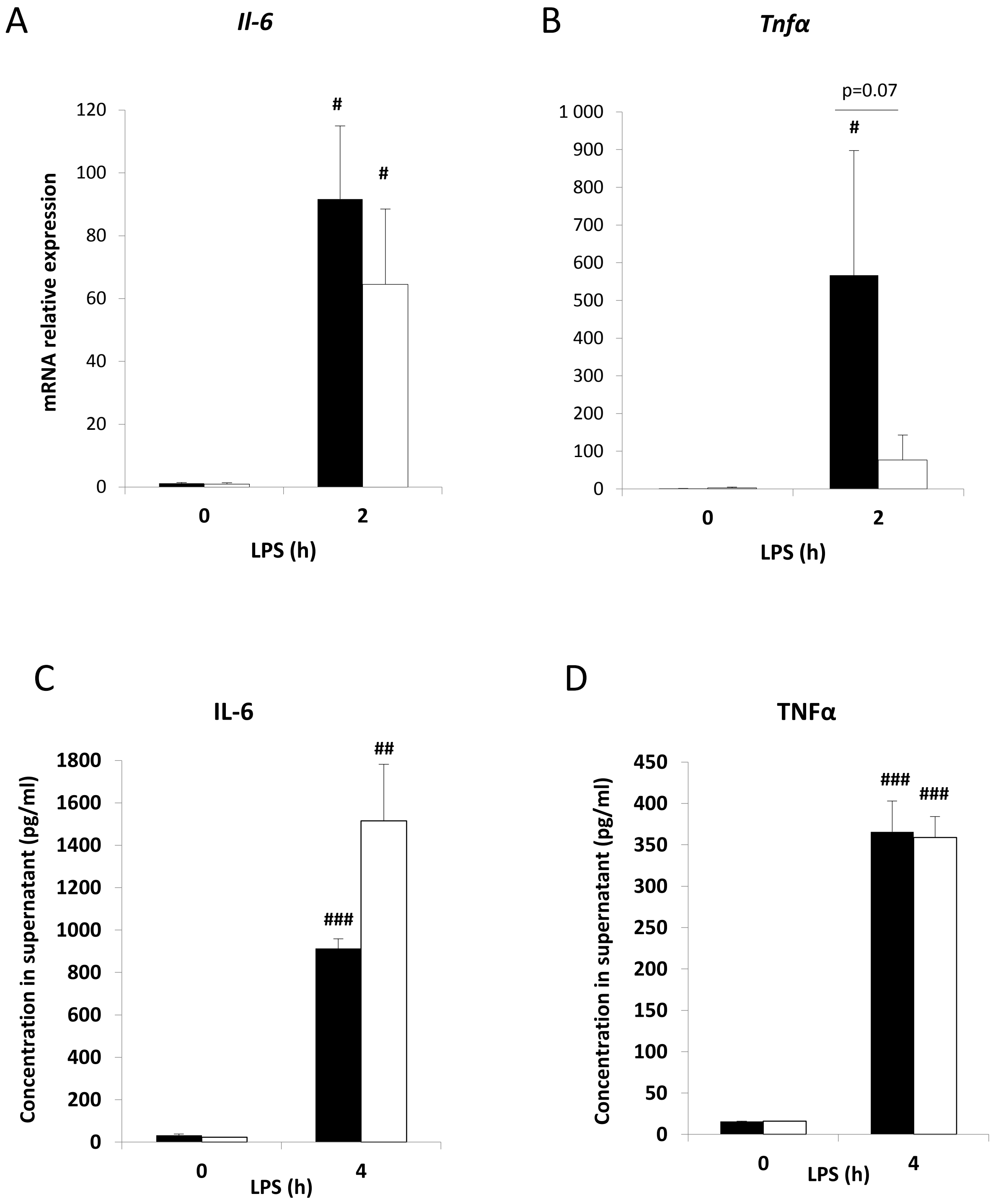
A
Il-6
mRNA relative expression
0
20
40
60
80
100
120
#
#
0
2
LPS (h)
B
Tnfα
p=0.07
#
0
100
200
300
400
500
600
700
800
900
1 000
0
2
LPS (h)
C
IL-6
Concentration in supernatant (pg/ml)
0
200
400
600
800
1000
1200
1400
1600
1800
###
##
0
4
LPS (h)
D
TNFα
Concentration in supernatant (pg/ml)
0
50
100
150
200
250
300
350
400
450
###
###
0
4
LPS (h)

Figure S4

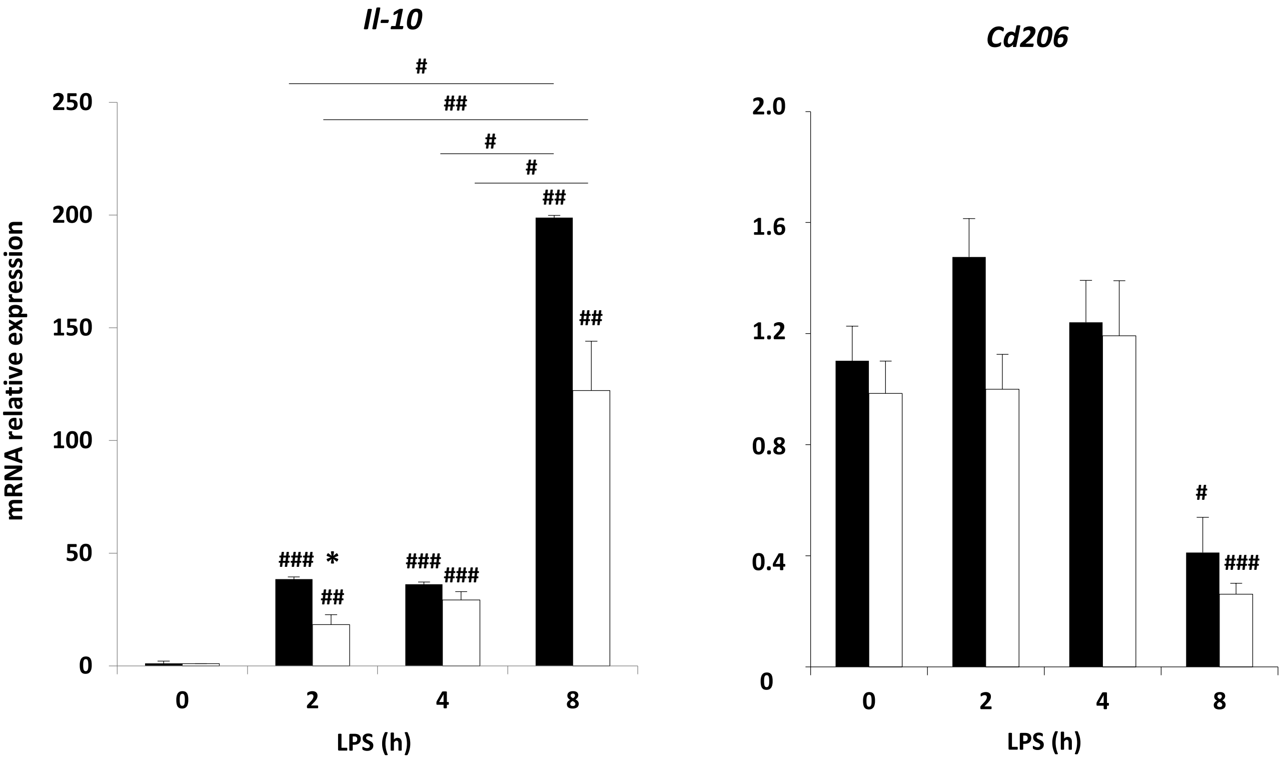

Il-10
Cd206
mRNA relative expression
250
200
150
100
50
0
2.0
1.6
1.2
0.8
0.4
0
#
##
#
#
##
##
*
##
###
###
#
###
0
2
4
8
LPS (h)
LPS (h)

Figure S5

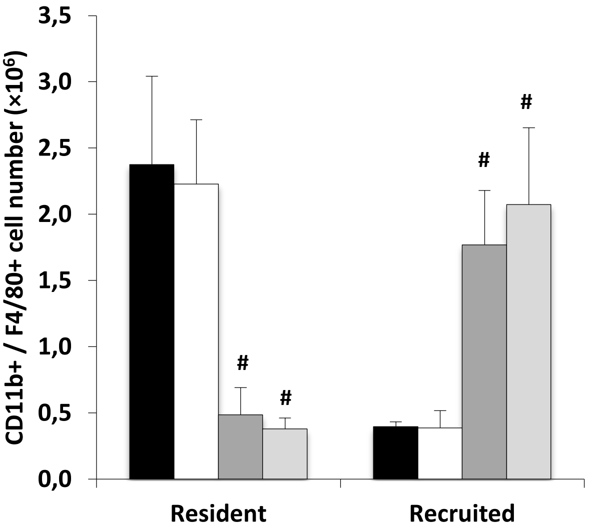
CD11b+ / F4/80+ cell number (×10^6)
3,5
3,0
2,5
2,0
1,5
1,0
0,5
0,0
#
#
#
#
Resident
Recruited

Figure S6

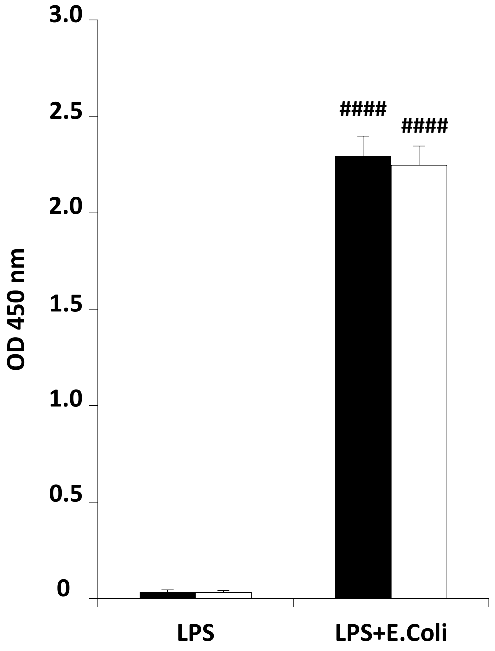

Figure S7

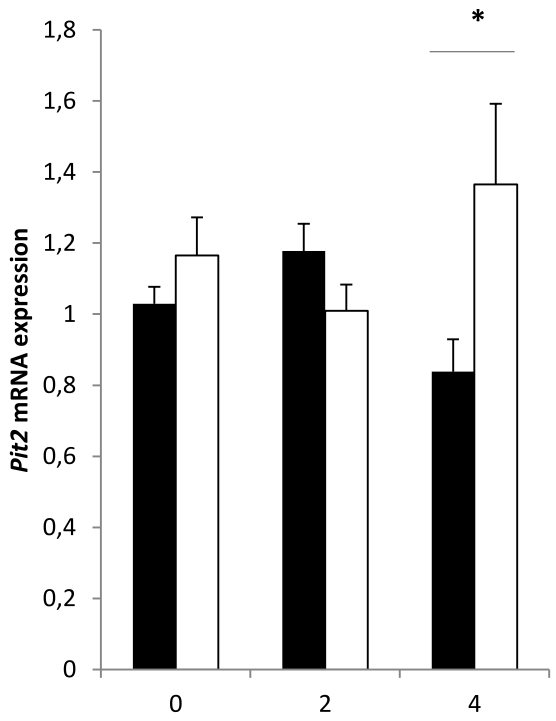

Figure S8

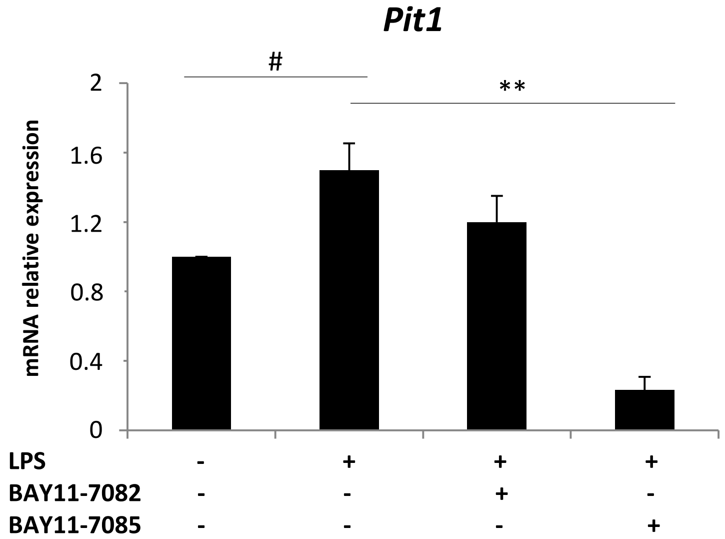

Pit1
#
**
mRNA relative expression
2
1.6
1.2
0.8
0.4
0
LPS - + + +
BAY11-7082 - - + -
BAY11-7085 - - - +

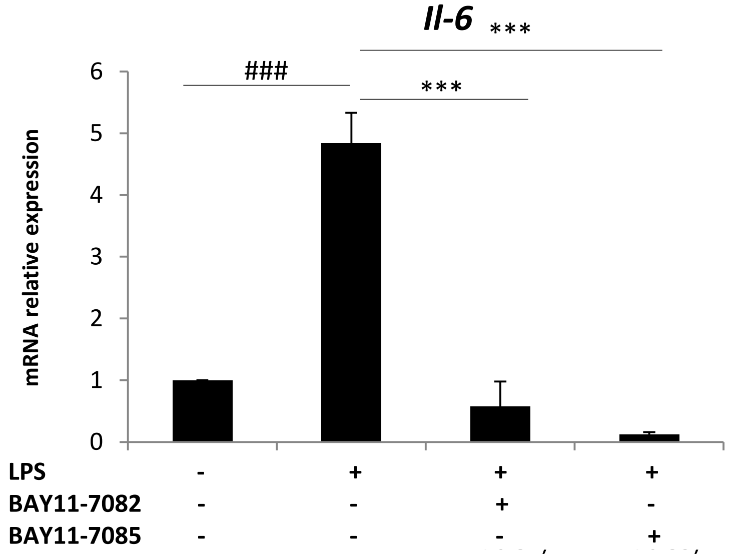

Il-6
***
###
***
mRNA relative expression
6
5
4
3
2
1
0
LPS - + + +
BAY11-7082 - - + -
BAY11-7085 - - - +

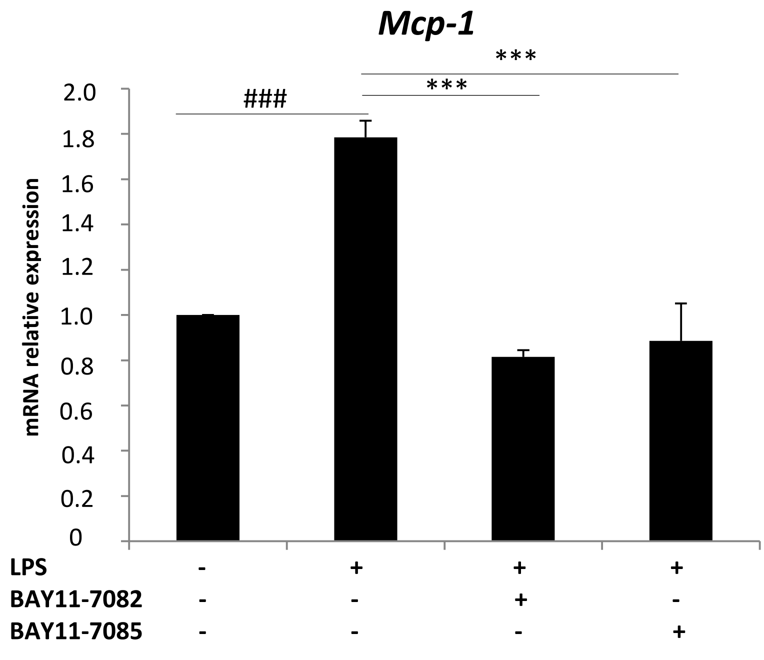

Mcp-1
***
###
***
mRNA relative expression
2.0
1.8
1.6
1.4
1.2
1.0
0.8
0.6
0.4
0.2
0
LPS - + + +
BAY11-7082 - - + -
BAY11-7085 - - - +

Supplementary figures S9. Uncropped images of western blots

Fig.5B

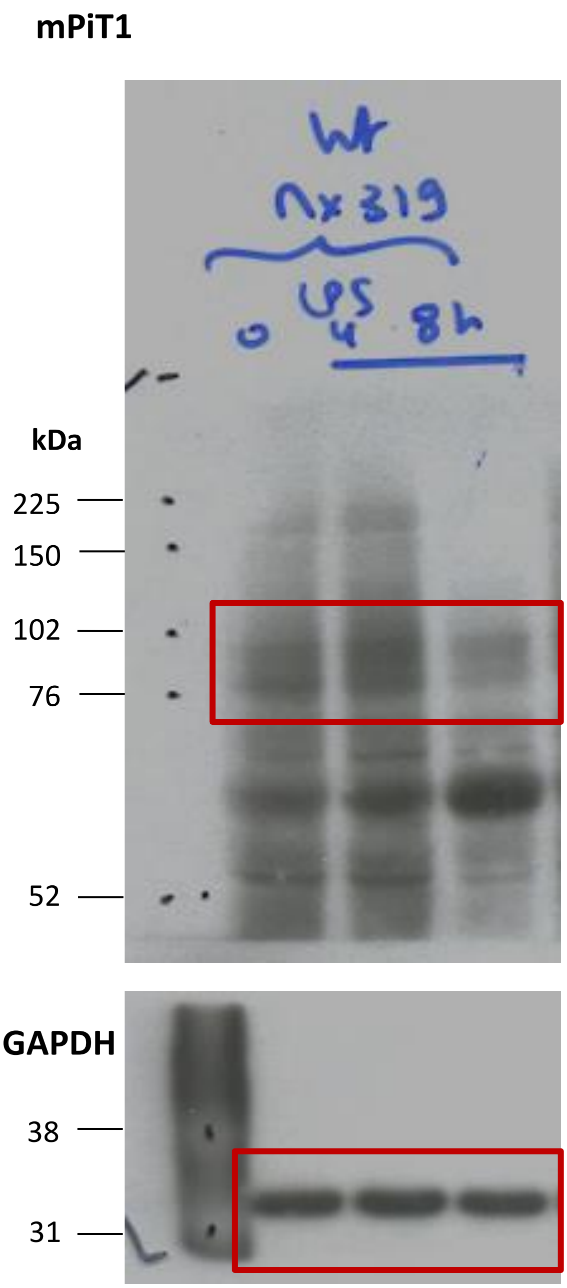

Fig.5E

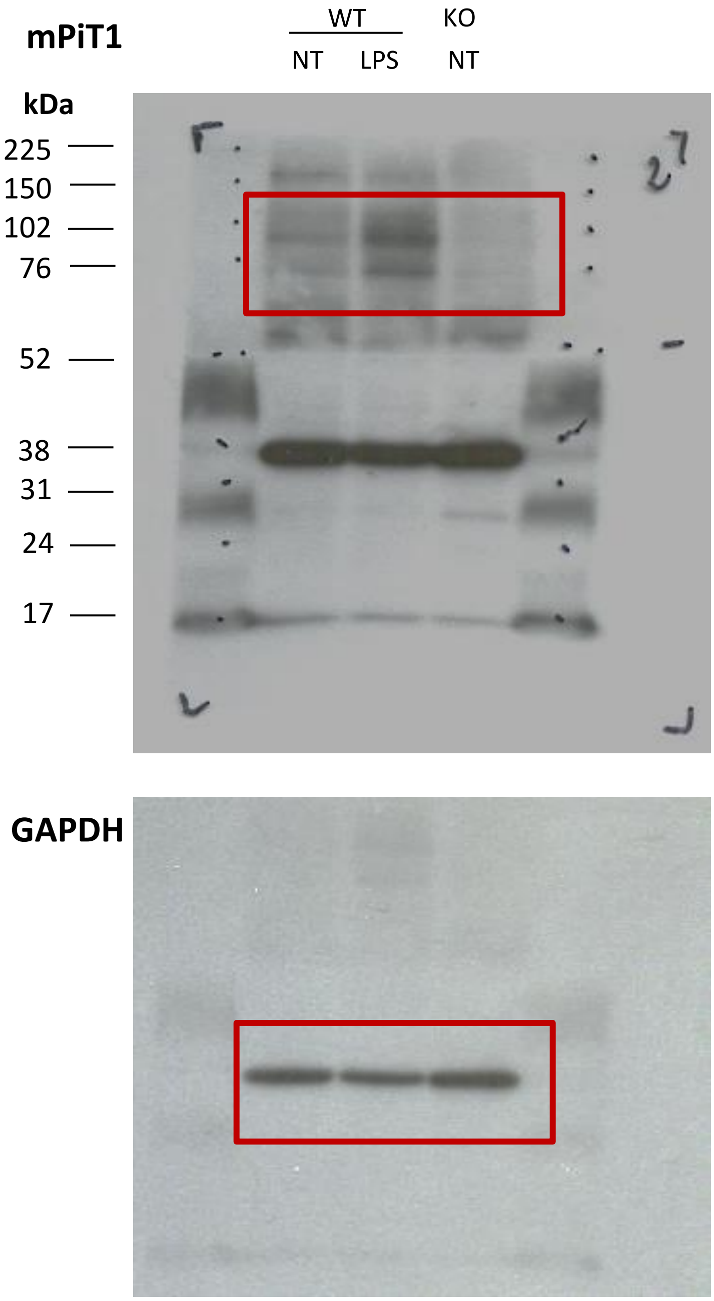

Supplementary figures S9. Uncropped images of western blots

Fig.6E

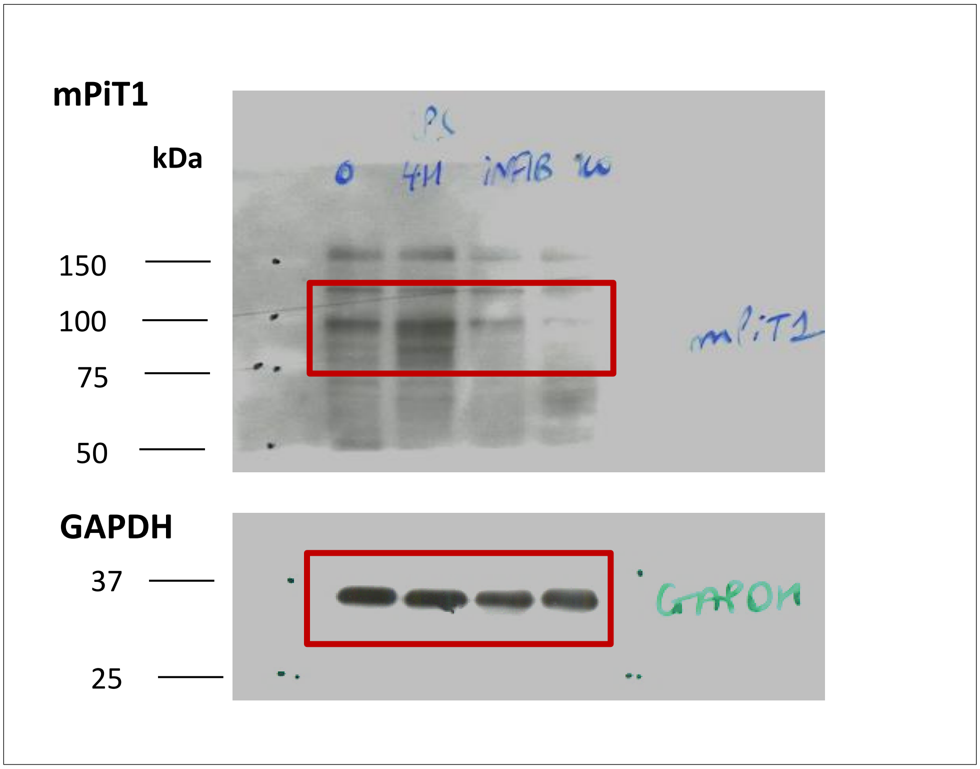

Supplementary figures S9. Uncropped images of western blots

Fig.7A

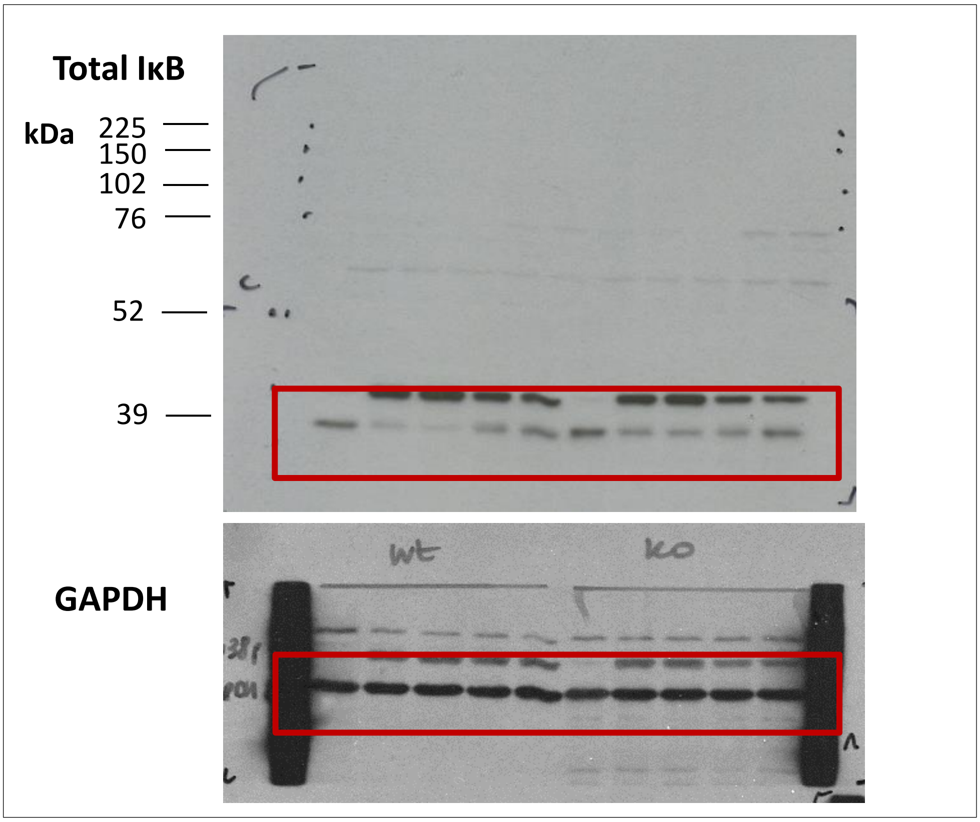

Fig.7B

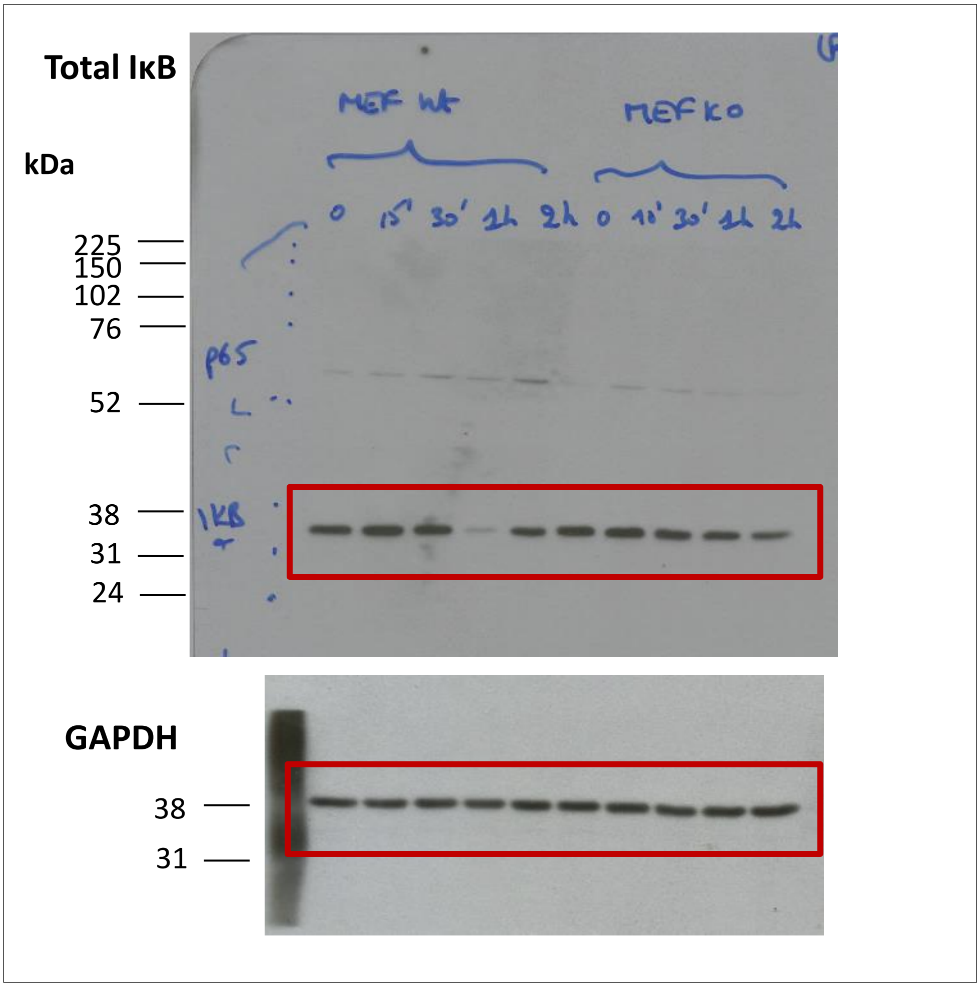

Supplementary figures S9. Uncropped images of western blots

Fig.7C

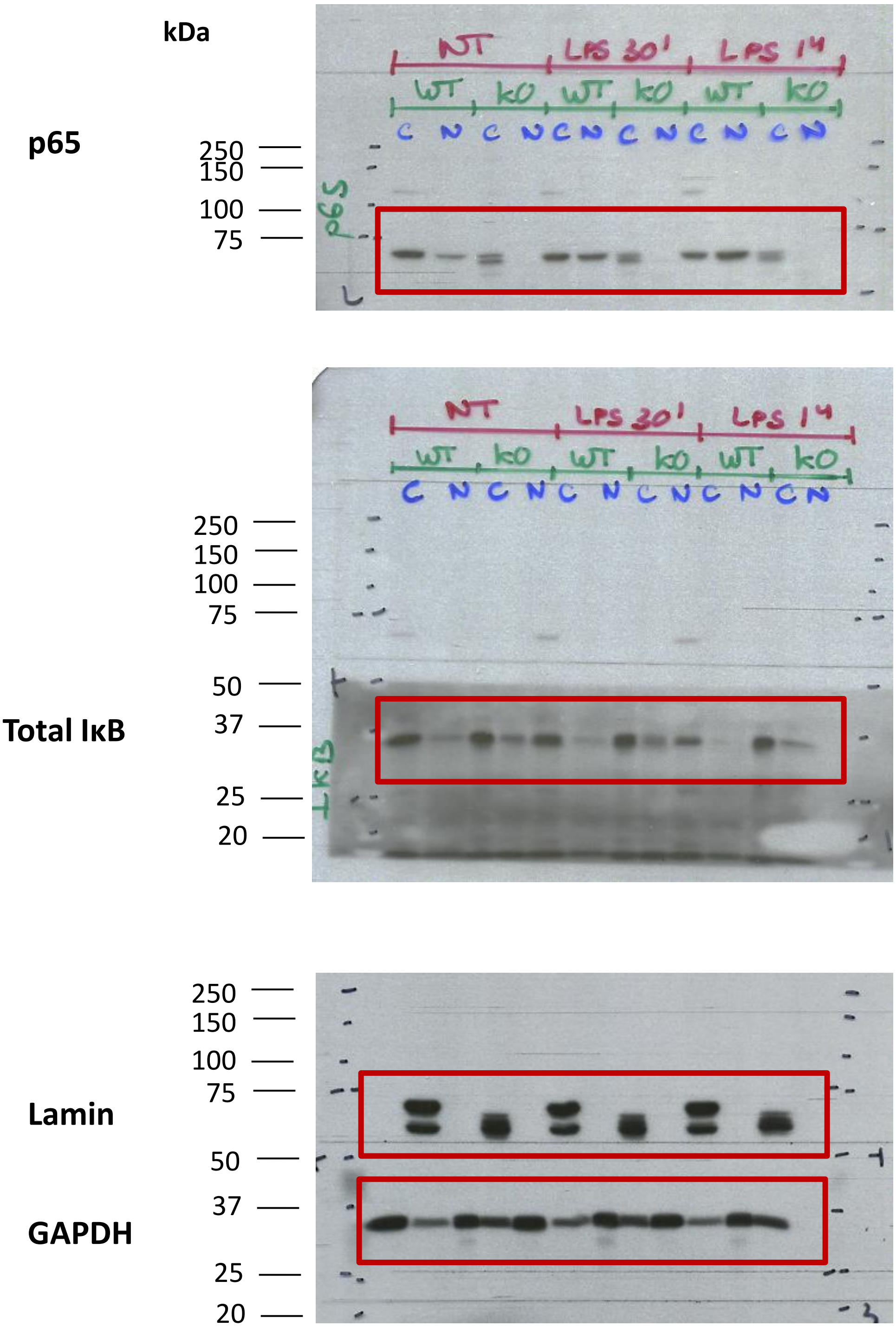

Supplement: Supplementary file 1 — Supplementary figures [file 41598_2018_37551_MOESM1_ESM.pdf]
